# The photomorphogenic factors UV-B RECEPTOR 1, ELONGATED HYPOCOTYL 5, and HY5 HOMOLOGUE are part of the UV-B signalling pathway in grapevine and mediate flavonol accumulation in response to the environment

**DOI:** 10.1093/jxb/erw307

**Published:** 2016-08-19

**Authors:** Rodrigo Loyola, Daniela Herrera, Abraham Mas, Darren Chern Jan Wong, Janine Höll, Erika Cavallini, Alessandra Amato, Akifumi Azuma, Tobias Ziegler, Felipe Aquea, Simone Diego Castellarin, Jochen Bogs, Giovanni Battista Tornielli, Alvaro Peña-Neira, Stefan Czemmel, José Antonio Alcalde, José Tomás Matus, Patricio Arce-Johnson

**Affiliations:** 1 Departamento de Genética Molecular y Microbiología, Facultad de Ciencias Biológicas, Pontificia Universidad Católica de Chile, Santiago, Chile; 2 Departamento de Fruticultura y Enología, Facultad de Agronomía e Ingeniería Forestal, Pontificia Universidad Católica de Chile, Santiago, Chile; 3 Centre for Research in Agricultural Genomics-CSIC-IRTA-UAB-UB (CRAG), Campus UAB, Bellaterra, Barcelona, Spain; 4 Wine Research Centre, University of British Columbia, Vancouver, BC, Canada; 5 Centre for Organismal Studies Heidelberg, University of Heidelberg, Heidelberg, Germany; 6 Department of Biotechnology, University of Verona, Italy; 7 Grape and Persimmon Research Division, Institute of Fruit Tree and Tea Science, NARO, Higashihiroshima, 73992494, Japan; 8 Laboratorio de Bioingeniería, Facultad de Ingeniería y Ciencias, Universidad Adolfo Ibáñez, Santiago, Chile; 9 Center for Applied Ecology and Sustainability, Santiago, Chile; 10 Weincampus Neustadt, DLR Rheinpfalz, Neustadt, Germany; 11 Departamento de Agroindustria y Enología, Facultad de Ciencias Agronómicas, Universidad de Chile, Santiago, Chile; 12 Quantitative Biology Center (QBIC), University of Tuebingen, Germany

**Keywords:** Binding, glycosyltransferase, MYBF1, network, photolyase, ripening, UVR8

## Abstract

Grapevine (*Vitis vinifera* L.) is a species well known for its adaptation to radiation. However, photomorphogenic factors related to UV-B responses have not been molecularly characterized. We cloned and studied the role of *UV-B RECEPTOR* (*UVR1*), *ELONGATED HYPOCOTYL 5 (HY5*), and *HY5 HOMOLOGUE* (*HYH*) from *V. vinifera*. We performed gene functional characterizations, generated co-expression networks, and tested them in different environmental conditions. These genes complemented the Arabidopsis *uvr8* and *hy5* mutants in morphological and secondary metabolic responses to radiation. We combined microarray and RNA sequencing (RNA-seq) data with promoter inspections to identify HY5 and HYH putative target genes and their DNA binding preferences. Despite sharing a large set of common co-expressed genes, we found different hierarchies for HY5 and HYH depending on the organ and stress condition, reflecting both co-operative and partially redundant roles. New candidate UV-B gene markers were supported by the presence of HY5-binding sites. These included a set of flavonol-related genes that were up-regulated in a *HY5* transient expression assay. We irradiated *in vitro* plantlets and fruits from old potted vines with high and low UV-B exposures and followed the accumulation of flavonols and changes in gene expression in comparison with non-irradiated conditions. *UVR1*, *HY5*, and *HYH* expression varied with organ, developmental stage, and type of radiation. Surprisingly, *UVR1* expression was modulated by shading and temperature in berries, but not by UV-B radiation. We propose that the UV-B response machinery favours berry flavonol accumulation through the activation of *HY5* and *HYH* at different developmental stages at both high and low UV-B exposures.

## Teaser

By performing molecular studies coupled to radiation experiments and *in silico* systems analyses, we have ascertained the role of the grapevine UV-B receptor and two HY5 homologues in regulating flavonol synthesis.

## Introduction

Ultraviolet B radiation (UV-B; 280–315nm) is a component of solar radiation and is harmful to all living organisms. Protective responses to UV-B in plants are triggered by perception and signalling systems whose components evolved early in the plant kingdom and are well conserved between species. They have been identified in sequenced plant genomes such as angiosperms, conifers, mosses, and algae (Tilbrook *et al.*, 2013). In the case of UV-B perception, these have been characterized in *Arabidopsis thaliana* (Heijde and Ulm, 2012) and in the unicellular algae *Chlamydomonas reinhardtii* (Tilbrook *et al.*, 2016).

Regardless of being a stress factor in plants, UV-B is also an environmental cue promoting several photomorphogenic and pathogen defence events during the plant’s life cycle (Kim *et al.*, 1998; Demkura and Ballaré, 2012). Because of this, two general signalling pathways are present in Arabidopsis for responding to UV-B radiation (Jenkins, 2009): a non-specific pathway, produced by high levels of radiation causing direct DNA damage and release of reactive oxygen species (ROS); and a specific signalling pathway, mediated by photomorphogenic components which respond to low levels of UV-A and UV-B radiation. The Arabidopsis ELONGATED HYPOCOTYL 5 protein (HY5) is a central mediator of UV-B protection and light photomorphogenic responses (Brown *et al.*, 2005; Stracke *et al.*, 2010). *HY5* is induced by UV RESISTANCE LOCUS 8 (UVR8), a UV-B receptor with specific tryptophan residues acting as intrinsic chromophores. UVR8 perceives radiation and dissociates from its non-active homodimer configuration (Rizzini *et al.*, 2011; Wu *et al.*, 2012). Following monomerization, UVR8 accumulates in the nucleus and interacts with CONSTITUTIVELY PHOTOMORPHOGENIC1 (COP1) (Cloix *et al.*, 2012), a WD40/RING protein that in dark or non-UV-inductive conditions targets HY5 for proteosome-dependent degradation (Saijo *et al.*, 2003). This interaction with UVR8 following the UV-B stimulus produces a switch from a repressing condition (with COP1 mediating the targeting of HY5 by the E3 ubiquitin ligase CUL4–DDB1) to a promoting state, due to the functional disassociation of the COP1–CUL4–DDB1 complex (Huang *et al.*, 2013).

Both photomorphogenic and non-specific responses to UV radiation share the accumulation of phenolic compounds derived from the activation of the phenylpropanoid pathway (Bornman *et al.*, 1997; Rozema *et al.*, 1997). The type of secondary metabolites that accumulate depends on plant species and UV spectra (A, B, or C), and comprises one or a mixture of compounds, such as flavonoids (e.g. anthocyanins and flavonols), stilbenes (resveratrol), cinnamate esters, and sinapoyl esters. Among the versatile range of functions that phenolic compounds possess, the most important for UV protection include their capacity to attenuate radiation by filtering (sunscreens), antioxidant activity capable of scavenging free radicals, and modulation of reactive oxygen signalling cascades involved in growth, development, and stress adaptation (reviewed by Hatier and Gould, 2009).

Plants improve their fitness under more severe environments by adjusting flavonoid accumulation to changes in radiation intensity and light quality (Tohge *et al.*, 2011). The plasticity in this response is reflected by the variation in flavonoid content found for the same species under different climate and agricultural conditions. Grapevine (*Vitis vinifera* L.) is a woody species often cultivated in Mediterranean climates with moderate to high UV-B radiation, with daily radiant exposures that generally range between 6 kJ m^−2^ d^−1^ (northern hemisphere) and 10 kJ m^−2^ d^−1^ (southern hemisphere) (Martínez-Lüscher *et al.*, 2013). Grapevines are highly adapted to solar radiation (Jug and Rusjan, 2012) due to a variety of long-term physiological responses (e.g. maintenance of the photosynthetic rate), mainly based on antioxidant enzyme activities and secondary metabolites involved in photochemical protective mechanisms (Martínez-Lüscher *et al.*, 2013). In fact, increased flavonoid content in response to UV-B has been reported in grapevine organs (Berli *et al.*, 2008; Pollastrini *et al.*, 2011; Martínez-Lüscher *et al.*, 2013, 2014; Liu *et al.*, 2015) although their relationship with UV-B perception and signalling is still not clear.

Considered as a relevant model for studying adaptive responses, grapevine has been examined through different approaches, in order to understand the effects of UV-B. Pontin *et al.* (2010) analysed the transcriptomic changes in leaves caused by a particular UV-B exposure (4.75 kJ m^−2^ d^−1^) provided at high and low fluence rates. These results demonstrated that general multiple stress pathways were the main activated responses in *Vitis*. However, a UV-B receptor and related signalling components were not identified. Additionally, Carbonell-Bejerano *et al.* (2014) analysed the late ripened berry skin transcriptome modulated by naturally occurring UV radiation using blocking filters in a mid-altitude vineyard. A few UV-B signalling pathway homologues were identified, but many were not modulated by UV-B filtering at late ripening stages. In addition, these were not characterized under inductive UV-B radiation conditions. In the present study, we have cloned and characterized the UV-B RECEPTOR 1 (UVR1) and two HY5 grape homologues (HY5 and HYH) as constituents of the grape UV-B response pathway. We hypothesize that the enhanced adaptation of grapevines towards radiation has been in part related to an extended response of HY5 and HYH to high radiation exposure, in addition to their photomorphogenic response to low UV-B. Our data suggest that HY5 and HYH play a complementary role in regulating flavonol synthesis in vegetative and reproductive organs of grapevine at different time points of development and different times after the UV-B stimulus.

## Materials and methods

### Cloning and relative expression quantification of grape *UVR1*, *HY5*, and *HYH* genes

We amplified the coding sequences (CDS) of *VviUVR1* (1338bp), *VviHY5* (510bp), and *VviHYH* (561bp) from cDNA samples isolated from different organs of cv. Cabernet Sauvignon. PCR fragments were amplified, cloned into pENTR™ Directional /SD/D-TOPO^®^ (Invitrogen), and sequenced.

RNA extraction, cDNA synthesis, and quantification of relative gene expression were carried out as in Matus *et al.* (2009). PCR conditions, primer sequences, and amplification efficiency coefficients are shown in Supplementary Table S1 at *JX*B online.

### Arabidopsis *uvr8* and *hy5* complementation analysis

*VviUVR1*- and *VviHY5*-containing vectors were recombined into pMDC32 and pK2GW7, respectively. The resulting Pro35S:*VviUVR1* and Pro35S:*VviHY5* expression vectors were introduced in *Agrobacterium tumefaciens* strain GV3101. The Arabidopsis null-mutant line *uvr8-6* (SALK_033468; Alonso *et al.*, 2003) and *hy5-215* (Oyama *et al.*, 1997) were transformed by floral dip. The T_3_ seeds derived from homozygous single insertions were grown as in Ulm *et al.* (2004). Hypocotyl length was measured as in Oravecz *et al.* (2006) with modifications: sterilized seeds (including wild-type) were placed on Corning^®^ square bioassay dishes (Sigma-Aldrich^®^) with Murashige and Skoog (MS) agar medium (2% sucrose) and stratified in darkness at 4 ºC for 3 d. Plates were then exposed to white light (120 µmol m^−2^ s^−1^) for 1h and returned to dark conditions for 23h. Plates were placed vertically for 6 d under continuous white light (4–5 µmol m^−2^ s^−1^) supplemented with Philips TL20W/01 RS SLV narrowband UV-B tubes (0.5W m^−2^ irradiance) or under continuous dark (covered with aluminium foil and polyester filters). As a control for no UV-B radiation, seedlings were exposed to light conditions described above but instead plates were covered with a clear polyester filter (100 μm, Interfilm). At the end of each treatment, plates were photographed and hypocotyl lengths were measured. The same seedlings were measured for flavonol accumulation as in Fornalé *et al.* (2014), with modifications: whole seedlings (100mg) were extracted in 1ml of 80% methanol at 4 ºC for 2h with gentle shaking. The mixture was centrifuged at 10 000rpm for 10min, 0.5ml of supernatant were taken to 2ml with methanol, and mixed with 0.1ml of aluminium chloride (10% w/v), 0.1ml of potassium acetate (1M), and 2.8ml of distilled water. After incubation at room temperature for 30min, absorbance at 415nm was measured. As a blank, the volume of 10% aluminium chloride was substituted with distilled water. Rutin was used as the calibration standard for quantifications. All experiments were performed with six biological replicates (*n*=6), using 15 seedlings (experimental units) for each replicate.

### HY5–GFP fusion and subcellular localization in agroinfiltrated tobacco plants

The CDS of *HY5* (excluding the stop codon) was cloned into pENTR™ Directional /SD/D-TOPO and recombined with pMDC84 to generate the vector *2xPro35S:HY5-GFP*. This was transformed into *A. tumefaciens* (strain GV3101) and infiltrated in *Nicotiana benthamiana* leaves. Three young leaves from 7-week-old plants (*n*=9) were infiltrated and kept in the greenhouse for 2 d. Three plants were irradiated with 0.2 W m^−2^ of UV-B for 2h under the same light conditions. Three plants were left inside a black box and kept in the same growth chamber. Leaves were collected at the same time of day, and screened for green fluorescent protein (GFP) using a laser-scanning confocal microscope (Olympus).

### Transcriptome expression and co-expression network analysis

The grapevine atlas microarray data set was obtained from Fasoli *et al.* (2012). Publicly available grapevine stress-related RNA sequencing (RNA-seq) sets were downloaded from the NCBI Sequence Read Archive (http://www.ncbi.nlm.nih.gov/sra). Raw FASTQ files were trimmed with trimmomatic v0.36 (Bolger *et al.*, 2014) using default parameters while ensuring that each read had an average quality score of 20 and a minimum length of 40 bases after trimming. Cleaned reads were aligned against the 12× grapevine reference genome PN40024 (Jaillon *et al.*, 2007) using bowtie2 v2.2.7 with default settings (Langmead and Salzberg, 2012). Read summarization was performed with a htseq-count v0.6.1 with default settings (Anders *et al.*, 2014). Differential expression analysis was performed with DESeq2 using a threshold of false discovery rate (FDR) <0.05 and absolute log2 fold change >1 to identify differentially expressed genes (Love *et al.*, 2014). Transcript abundance was determined using the variance-stabilized transformation procedure of DESeq2.

Gene co-expression analysis was performed on the atlas and RNA-seq data sets using the mutual rank and PCC as co-expression similarity indexes (Obayashi and Kinoshita, 2009). Additional gene co-expression mining from microarray stress data sets was performed with VTCdb v2.1 (Wong *et al.*, 2013). The top 300 co-expressed gene targets with grapevine *HY5* and *HYH* ‘guide’ genes were retained from the three data sets and merged into a final aggregate network. Enrichment of Gene Ontology (GO) categories within co-expressed genes was determined using g:Profiler (http://biit.cs.ut.ee/gprofiler/) at a significance threshold of FDR <0.05.

### Genome-wide survey of HY5 and related transcription factor-binding sites (TFBS)

Grapevine promoter sequences (1kb upstream of the transcription start sites, TSS) were downloaded from EnsemblPlants (http://plants.ensembl.org/index.html). Promoter scanning of previously determined high affinity binding sites of Arabidopsis HY5 and soybean STF1 (HY5 homologue) proteins (C-box, C/A-box, and C/G-box; Song *et al.*, 2008) and UV-B-specific motifs (E-box and T/G-box; Binkert *et al.*, 2014) was conducted. The consensus sequences and individual octamer combinations derived from these consensus (83 in total) were scanned in promoter regions (on both + and – strands) of the entire genome and co-expressed target genes as described previously (Savoi *et al.*, 2016). The position bias of TFBS within promoter regions (expressed as *Z*-score) was determined based on a uniform distribution model (Ma *et al.*, 2013). Enrichment for the presence of motifs in promoters of co-expressed genes was determined by a hypergeometric test. Statistical significance of total *cis*-regulatory element (CRE) occurrences in promoters of co-expressed genes was derived from a permutation test using randomized promoters of similar size. An empirical *P*-value was determined from 1000 permutations. In parallel, the fold enrichment between observed and expected (random) numbers of motif occurrences was determined. Motifs were considered significantly enriched at *P*<0.05 (or *P*<0.01, strict) in both tests. All statistical tests were performed in R (http://www.bioconductor.org/).

### Generation of the VP64–HY5 construct and agroinfiltration in grapevine plantlets

Four copies of the herpes simplex virus VP16 transactivation domain (named VP64) were fused to the N-terminus of HY5 by gene synthesis (Epoch Life Science). The product was subcloned into pDONR207 and recombined into pMDC32 (*2xPro35S*:*VP64-HY5*), and used to transform *A. tumefaciens* strain C58C1. As a negative control, the same *Agrobacterium* strain was transformed with an empty vector. Twelve *in vitro* grapevine plantlets of cv. Sultana were kept in low light conditions during the whole experiment (before and after the agroinfiltration). Six plants were immersed in each bacterial suspension (*VP64-HY5* or empty vector) and vacuum infiltrated (2×2min at 90 kPa) as described in Cavallini *et al.* (2015). After agroinfiltration, plantlets were rinsed with sterile water and allowed to recover (*in vitro* conditions) for 5 d before collecting leaves and proceeding with RNA extraction and cDNA synthesis. *HY5* expression was calculated as described previously. For expression level calculations of putative target genes, we selected the three plantlets characterized by high and comparable expression levels of the transgene. Similarly, three lines showing low and comparable *HY5* expression were selected from the group of negative control plants.

### Low UV-B exposure treatments in vegetative tissues and flavonol content analysis

*In vitro* grapevine plants were exposed for 6h to UV-B radiation as in Cavallini *et al.* (2015). Plants were immediately taken to the *in vitro* chamber and two different sampling procedures were followed: (i) immediately after the treatment ended (6h, for gene expression analyses and (ii) at 48h and 96h during the recovery stage (for flavonol quantification, as described above).

In a second experiment, dormant hardwood cuttings were placed in a hydroponic system with sterile Perlite and kept at 25 °C with a light intensity of ~100 μmol m^–2^ s^–1^ and a 9h light cycle. Under these conditions, apical buds burst after 10 d and roots emerged after 4 weeks. Nineteen days later, cuttings were transferred to 23 °C, 4% UV-B, and 30% UV-A light (18W, 6500K) conditions, maintaining the 9h light cycle. Control plants grew under the same conditions but without UV-B. Leaves were harvested at 0, 10, 24, 48, and 72h after the onset of light+UV-B exposure, and immediately frozen in liquid nitrogen (*n*=9).

### Low and high UV-B exposure treatments in fruits and flavonol content analysis

The experiment was carried out during 2011–2012 and 2012–2013 seasons inside a phytotron. Six years prior to the experiment, 9-year-old cv. Cabernet Sauvignon plants were carefully uprooted from a commercial vineyard in Santiago, Chile (latitude 33º36'17''S, longitude 70º36'20''W) and transferred to 70 litre pots with a copper hydroxide-based paint previously applied on the inside. Soil substrate consisted of 2 vols of leaf compost, 2 vols of sieved soil, and 1vol. of sand. After 3 years growing under partial shade, six plants were transferred to a UV-free greenhouse with temperature control, simulating a normal daily cycle under growing conditions. Two UV-B-inductive treatments were implemented at ~5 d after fruit set: in the 2011–2012 season, plants were exposed to high UV-B irradiance and in 2012–2013 to a low UV-B irradiance (both contrasted to control ‘filtered/non-irradiated’ conditions). UV-B radiation was proportioned using a TL20W/12 RS SLV tube (68% UV-B and 32% UV-A, Philips) suspended 90cm (high UV-B) and 120cm (low UV-B) facing bunches of grapes in order to produce different irradiances by variation with distance. Incident minimum and maximum UV-B irradiance from solar radiation perceived by commercial vineyards in summer in the morning (9:30h) and midday (12:00h) is typically 0.067W m^−2^ and 0.358W m^−2^, respectively. Therefore these contrasting UV-B irradiances were mimicked under greenhouse conditions. UV-B exposure levels of ~0.3W m^−2^ and ~0.1W m^−2^ for high and low irradiance were applied daily for 5h and 10h, respectively, aiming to achieve a similar total daily biologically effective UV-B exposure (UV-B_BE_ 5.4 kJ m^−2^ d^−1^) for both treatments. Grape clusters were treated in two ways each season: (i) ‘filtered cluster’ as a non UV-B-irradiated condition, where clusters were covered with a polyester filter (100 μm clear polyester) absorbing 100% of UV-B without significantly affecting photosynthetically active radiation (PAR); and (ii) a non-filtered treatment (+UV-B). Tubes were covered with cellulose acetate filters excluding wavelengths lower than 280nm (UV-C radiation). UV-B radiation was measured using a VLX-3.W UV radiometer equipped with a CX-312 UV-B sensor (Vilber Loumart, Germany). Sample collection was distributed at five stages of grapevine development: –3, 0, 3, 6, and 9 weeks from veraison (WAV). A total of 18 berries were sampled from nine grape clusters (*n*=3, three bunches per plant and two berries per cluster). Weeks –3, 0, 3, and 6 were used for RNA extraction and gene expression quantification by real-time PCR (qRT-PCR), whereas samples at week 9 were used for HPLC analysis at the final stage of maturation. Berries were immediately peeled and deseeded after collection, and skins were frozen in liquid nitrogen and then stored at –80 ºC until required for RNA extraction. Veraison was determined as the time at which clusters were 30–50% coloured and sugar concentration reached 5º Brix. HPLC quantification of flavonols was conducted as described by Matus *et al.* (2009).

## Results and Discussion

### Conservation of grape *UVR1* and *HY5* homologues

The DNA sequence of *AtUVR8* (GenBank accession no. AF130441) was used in a BLAST search against the grapevine PN40024 12Xv1 genomic prediction. Only one gene model, with 1338bp (12 exons), was found (*VIT_07s0031g02560*) and its CDS was amplified from cv. Cabernet Sauvignon and named *UV-B RECEPTOR 1* (*VviUVR1*, GenBank accession no. JX867716). VviUVR1 is a 445 amino acid long protein, sharing 99.6% identity with the predicted sequence from cv. Pinot Noir. VviUVR1 possesses seven Regulator of Chromosome Condensation (RCC1) domains and 14 highly conserved tryptophan residues (Supplementary Fig. S1A). Tryptophan is responsible for most of the absorbance of near UV light (Creed *et al.*, 1984). In AtUVR8, Trp285 acts as an intrinsic chromophore (Rizzini *et al.*, 2011). In VviUVR1, W285 is present. Phylogenetic analysis revealed a close relationship of VviUVR1 to other dicot UVR8-like proteins (Supplementary Fig. S1B).

Using *AtHY5* (AB005456) and *AtHYH* (NM_180274) as queries, we found *VIT_04s0008g05210* and *VIT_05s0020g01090* gene models. These were described as the only members of the basic-leucine zipper (bZIP) subgroup G (group H in Arabidopsis; Jakoby *et al.*, 2002) and were identified as *VvibZIP10* and *VvibZIP15*, respectively (Liu *et al.*, 2014). We cloned their CDS from cv. Cabernet Sauvignon berry skin cDNA and named them *VviHY5* and *VviHYH* (GenBank accession no. KF356359 and KJ423106, respectively). The VviHY5 protein is 169 amino acids in length and is identical to the corresponding predicted gene model in PN40024. However, VviHYH (186 amino acids) shared only 63% identity with its predicted gene model due to an incorrect annotation. The correct sequence with a complete motif 1 (ESDEELx_2_VP[DE][MF][GE]) was confirmed by our cloning. We observed a high degree of conservation in the first four residues of the COP1 interaction core sequence (VPE Φ^G^/_E_, Holm *et al.*, 2001; Song *et al.*, 2008) of VviHY5, VviHYH, and their homologues from other plant species (Supplementary Fig. S2).

### Functional analysis of VviUVR1 and VviHY5

**Fig. 1.**
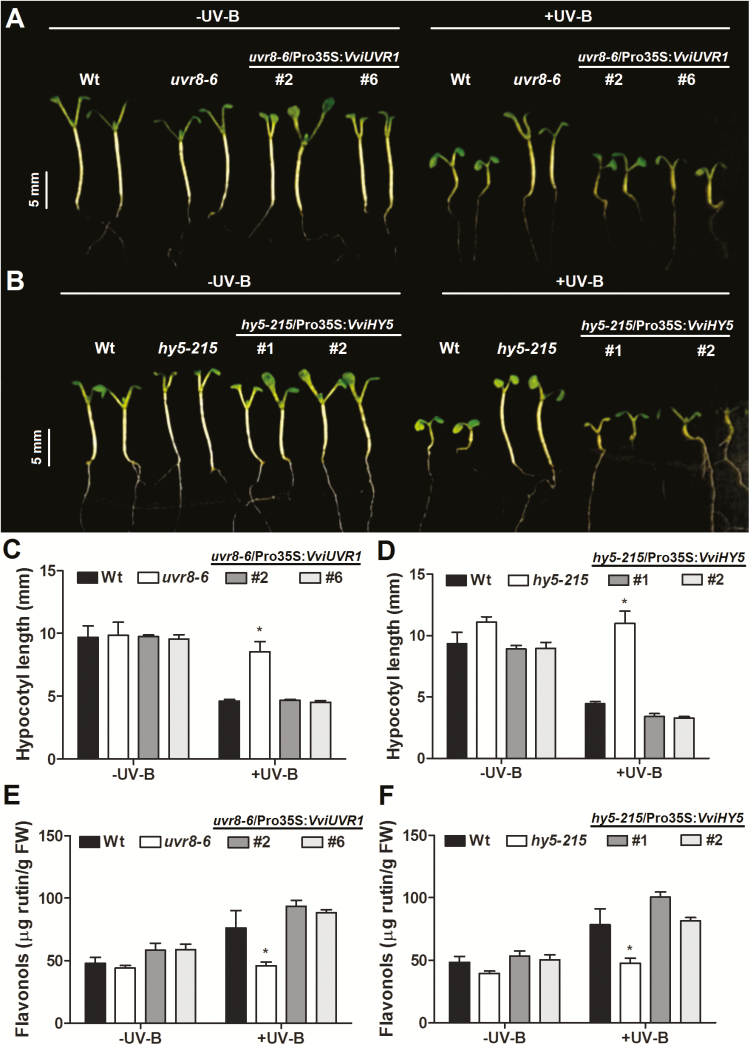
Transgenic expression of *VviUVR1* and *VviHY5* in Arabidopsis complements the *uvr8* and *hy5* mutant UV-B phenotypes. (A–D) Restored UV-B-induced hypocotyl growth inhibition in *UVR1* and *HY5* complementation lines. Wt (Col-0), *uvr8-6* mutant, *hy5-215* mutant, and *UVR1* and *HY5* complementation lines were grown under white light with or without supplementary UV-B radiation. (E, F) Total flavonol accumulation in 6-day-old seedlings grown under white light with or without supplementary UV-B. Error bars represent the SD (*n*=6 plates with 15 seedlings each). Asterisks indicate statistical significance. (This figure is available in colour at *JXB* online.)

To confirm the role of VviUVR1 as a UV-B receptor, we expressed its CDS under the control of the *Cauliflower mosaic virus* 35S promoter in Arabidopsis *uvr8-6* mutant plants (SALK_033468). Six independent hygromycin-resistant lines were confirmed by RT-PCR (Supplementary Fig. S3A). Transgenic lines (*uvr8*-6/Pro35S:*VviUVR1*) were compared with wild-type (Col-0) and *uvr8-6* mutant plants by measuring hypocotyl elongation and flavonol accumulation in white light±UV-B or dark conditions as decribed by Kliebenstein *et al.* (2002) (Fig. 1; Supplementary Fig. S4). The constitutive expression of *VviUVR1* in the *uvr8* background restored the inhibition of hypocotyl growth in white light plus UV-B (Fig. 1A–C). Likewise, flavonol accumulation was restored and enhanced in the *uvr8*-6/Pro35S:*VviUVR1* lines upon radiation (Fig. 1E). These results demonstrate that VviUVR1 plays the same role as AtUVR8 in UV-related photomorphogenesis and in flavonoid synthesis in response to UV-B.

We overexpressed *VviHY5* in Arabidopsis *hy5-215* mutant plants. Four independent transgenic lines were generated and checked by PCR (Supplementary Fig. S3B). In all lines tested (*hy5-215*/Pro35S:*VviHY5*), the UV-B-induced inhibition of hypocotyl growth was restored (Figs. 1B, D). Flavonol accumulation in response to UV-B was also restored in VviHY5-complemented lines (Fig. 1F). Taken together, we confirmed that VviUVR1 and VviHY5 are the functional orthologues of AtUVR8 and AtHY5 and are able to restore the UV-B signalling pathway in Arabidopsis. Due to the high homology of VviHYH to AtHYH and also VviHY5, we propose HYH as a functional homologue of HY5.

**Fig. 2.**
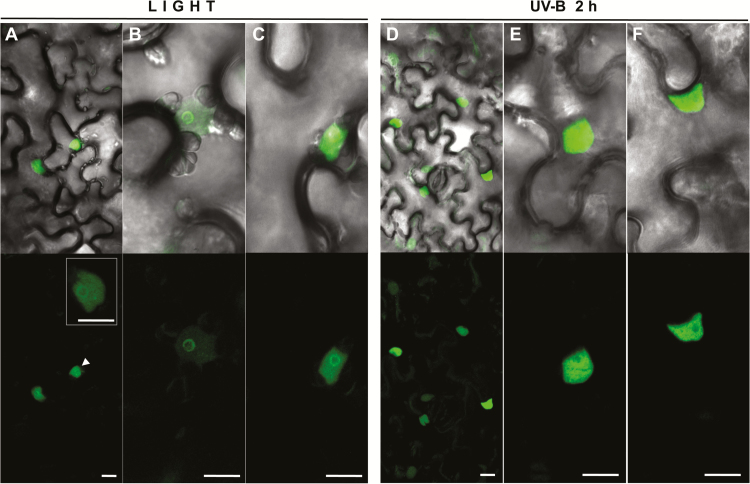
VviHY5–GFP subcellular localization in agroinfiltrated tobacco leaves. Two days after infiltration, plants were kept in light (A–C) or transferred to a chamber with light supplemented with UV-B for 2h (D–F). Light-field images are merged with the GFP filter. Bars represent a scale of 5 µm. The insert in (A) shows nucleolar peripheral localization and a nuclear spleckle in the nucleus indicated with an arrowhead. (This figure is available in colour at *JXB* online.)

Photomorphogenic factors are often regulated by transcriptional and post-translational mechanisms. We agroinfiltrated *N. benthamiana* plants with a 35Spro:*VviHY5-GFP* fusion construct and followed HY5 subcellular localization (2 d after infiltration) in light, light supplemented with UV-B, and complete dark growth conditions. Under normal light, HY5–GFP was localized in the nucleus of tobacco epidermal cells (Fig. 2A–C). Interestingly, in most of the fluorescent nuclei, we found preferential peripheral nucleolar localization, sometimes even brighter than in the rest of the nucleus. In a few cases, some discrete nuclear speckles were also observed. After 2h of UV-B irradiation, the GFP signal was more diffuse in the entire nucleus and excluded from the nucleolus (Fig. 2D–F). In dark conditions, GFP fluorescence signal was barely detectable.

We show that VviHY5 localization changes depending on light composition. The perinucleolar localization of HY5 in normal light conditions has not been reported before in other plant species and could represent localized HY5 protein modification or degradation mediated by the COP1 machinery. This mechanism has also been suggested when observing AtHY5 and AtHYH being co-localized with AtCOP1 in nuclear speckles following their co-transformation (Ang *et al.*, 1998; Holm *et al.*, 2002). Thus, both speckles and the nucleolus periphery could represent sequestering sites for arresting HY5 function, which are later abolished after an inductive UV-B stimulus.

### Gene expression patterns of *UVR1*, *HY5*, and *HYH*

**Fig. 3.**
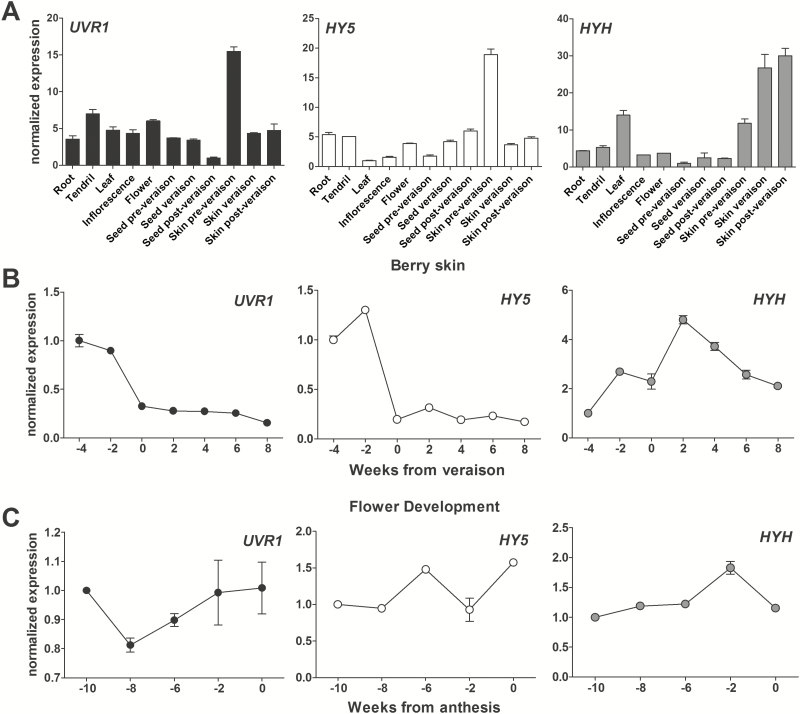
Comparison of *UVR1*, *HY5*, and *HYH* expression levels in grapevine organs of field-grown plants of cv. Cabernet Sauvignon. Expression in (A) all organs tested, (B) berry developmental stages, and (C) inflorescence development. Expression levels were normalized against the tissue of lowest expression in (A) and the expression at the first developmental stage in (B) and (C). SDs are the result of three independent replicates.

As UVR1 and HY5 are functional members of the UV-B signalling pathway, we were interested in determining if their expression was also related to their function in different organs of the grapevine. We obtained the spatio-temporal expression of *UVR1*, *HY5*, and *HYH* in different organs and developmental stages of field-grown grapevine plants (Fig. 3). *UVR1* expression was lowest in late-stage seeds and highest, in decreasing order, in green-stage berries, flowers, and tendrils (Fig. 3A). During berry development, this high expression was limited to early developmental stages (at –4 and –2 WAV) (Fig. 3B). A similar expression pattern was reported by Liu *et al.* (2015) in the white skin of cv. Sauvignon Blanc grape berries. *UVR1* expression during flower development was maintained with almost no variation (Fig. 3C).

Similarly to *UVR1*, *HY5* was highly expressed at green-stage berries and its lowest expression was found in leaves and early-stage seeds (Fig. 3A). During berry development, *HY5* expression peaked at –2 WAV (Fig. 3B). In contrast, *HYH* was highly expressed at veraison and post-veraison, followed by leaves (Fig. 3A). Its expression peaked after veraison (2 WAV) and, though it decreased thereafter, it was always higher than at the very early stage (Fig. 3B). A relatively stable expression of both *HY5* and *HYH* was observed during inflorescence development (Fig. 3C).

We compared our results with the expression of these genes found in the previously published global expression atlas of cv. Corvina (Fasoli *et al.*, 2012; Supplementary Fig. S5). *UVR1* was predominantly expressed in some floral organs, berry skin, tendril, rachis, leaf, and bud. It was down-regulated in seeds, roots, and at post-harvest withering stages of berry pericarp, flesh, and skin. In berry flesh, a marked down-regulation of *UVR1* was detected at veraison and ripening stages, while a moderate expression was observed throughout berry skin development. *HY5* was expressed in floral buds (mostly before flowering), berry skins, and woody stems. Like in cv. Cabernet Sauvignon, the *HY5* expression pattern was very similar to that of *UVR1* in berry skin development where the highest mRNA levels were found at the green berry stage, with a general decrease thereafter. Transcript accumulation of *HYH* was more divergent from that of *UVR1* and *HY5*, especially throughout berry development. *HYH* had a higher expression in vegetative tissues such as leaves and tendrils, although it was induced similarly to HY5 in stems.

### Identification of *HY5* and *HYH* co-expressed genes (CEGs) and search for potential targets

As HY5 and HYH show certain differences in expression, we further tested if their regulatory networks had also diverged in their composition, by performing a systems-oriented analysis of co-expression and presence of TFBS. Gene co-expression networks (GCNs) are becoming increasingly used to infer gene function, and set common pathways and putative targets for transcription factors (Proost and Mutwil, 2016). In grapevine, a few cases have being adopted to understand how gene networks govern berry development and composition (Palumbo *et al.*, 2014; Wong *et al.*, 2016)

To define *HY5* and *HYH* community co-expression networks, we integrated networks constructed from stress-related RNA-seq experiments (listed in Supplementary Table S2) and microarray data from the grapevine gene expression atlas (Fasoli *et al.*, 2012) together with a previously described stress-dependent network (VTCdb; Wong *et al.*, 2013). In addition, we scanned the promoter regions of all grapevine genes (29 971 including HY5/HYH CEGs) for the presence of HY5-binding elements. The main purpose of these analyses was to define the overlap of putative targets between HY5 and HYH, and also to determine the extent of the stress- and developmentally related gene networks from these two transcription factors. This analysis led us to compare up to 300 CEGs for each stress and developmental network (six networks in total). Several studies have shown that CEGs within the top 300 generally provide a reasonable ceiling for biological validation while maintaining statistical significance (Wong *et al.*, 2014; Aoki *et al.*, 2016).

Our analysis clearly shows that VviHY5 and VviHYH are part of a radiation response pathway, as several genes related to DNA repair, heat shock chaperones, and light signalling responses were among the most highly co-expressed (Supplementary Table S3). We observed a partial overlap between *HY5* and *HYH* networks, with 24% (72 genes) and 37% (111 genes) of the top 300 CEGs being shared in the tissue atlas and stress-related RNA-seq data sets, respectively. Consistently, the top CEG lists of *HY5* and *HYH* stress-dependent networks obtained from VTCdb have 35% overlap (82 genes). *HY5* and *HYH* were listed in their counterpart networks, supporting a co-operative role as observed for AtHY5 and AtHYH in the Arabidopsis UVR8-dependent pathway (Brown and Jenkins, 2008). GO analysis revealed that both *HY5* and *HYH* data sets were enriched in photosynthesis, abiotic stimulus, and lipid metabolic processes (Supplementary Fig. S6). A closer inspection of these data sets also showed that both *HY5* and *HYH* stress data sets were highly enriched in light, heat, and radiation terms, as well as oxidative stress, protein folding, and cuticle development (Supplementary Table S4). Additionally, *HYH* was highly enriched with terms related to response to red/blue light and pigment metabolic processes.

**Fig. 4.**
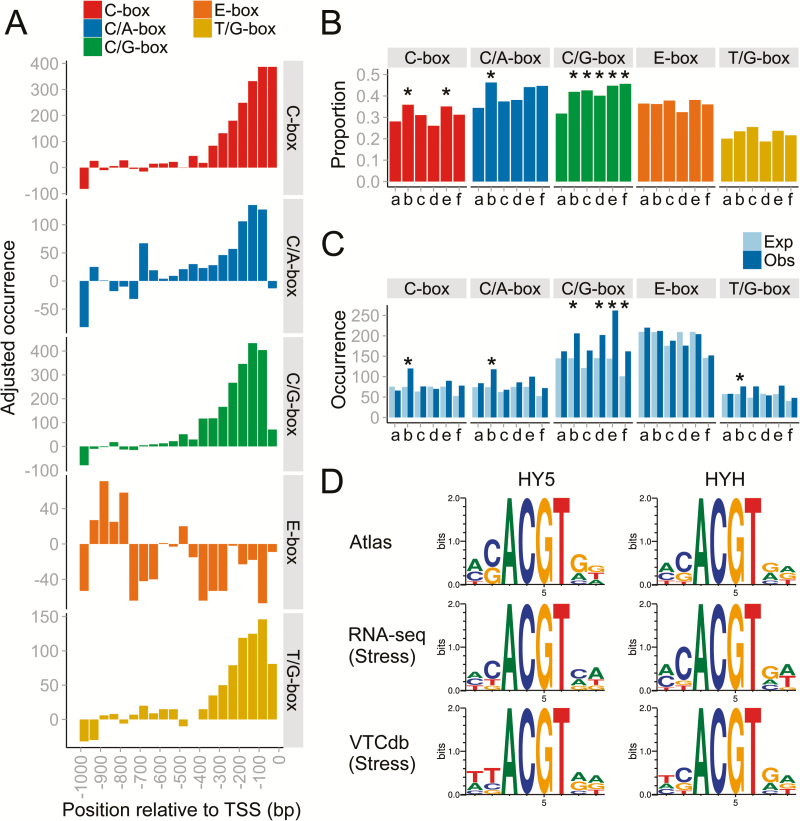
Genome-wide analysis of predicted HY5 and UV-B response *cis*-regulatory elements (CREs) in grapevine promoters. (A) Distribution plots of HY5 and UV-B response CREs in grapevine promoters (1kb upstream of the TSS). Each bin represents the total motif occurrence in 50 promoter bases, adjusted for the baseline occurrence (average motif occurrence between the –500 and –1000bp region). The baselines of the C-box, C/A-box, C/G-box, E-box, and T/G-box are 490, 798, 600, 709, and 333, respectively. Motif *Z*-scores for the C-box, C/A-box, C/G-box, E-box, and T/G-box are 6.4, 1.6, 5.8, –0.7, and 2.9 respectively. (B) Frequencies (in proportion) of highly co-expressed genes with *HY5* and *HYH* inferred from various data sets (atlas, stress-related RNA-seq, and stress-related VTCdb) containing the relevant CRE in their promoter region. Strong enrichment of the CRE based on the differences in proportions following a hypergeometric distribution is indicated as * (*P*<0.01). (C) The total number of CRE occurrences (Obs) in promoter regions of *HY5* and *HYH* co-expressed genes and randomized promoters of similar size (Exp). Statistically significant CRE observations at *P*<0.01 (based on 1000 permutations) are indicated by asterisks. (D) The sequence logo for grapevine *HY5* and *HYH* inferred from degenerate binding sites enriched in various data sets. a, HY5 (Atlas); b, HY5 (Stress RNA-seq); c HY5 (Stress VTCdb); d, HYH (Atlas); e, HYH (Stress RNA-seq); f, HYH (Stress VTCdb). (This figure is available in colour at *JXB* online.)

We further searched for HY5/HYH DNA binding preferences. Song *et al.* (2008) performed a comprehensive study to determine AtHY5 and STF1 (Soybean homologue of HY5) binding sites (TFBS), determining three major consensus sequences: HBACGTCD [C-box], (A/C/T)(C/G/T)ACGTC(A/G/T); HBACGTGD [C/G-box], (A/C/T)(C/G/T)ACGTG(A/G/T); and HBACGTAD [C/A-box], (A/C/T)(C/G/T)ACGTA(A/G/T). These motifs share the ACGT core typically found in light-responsive ACGT-containing elements (ACEs). In addition, a recent study by Binkert *et al.* (2014) showed that both HY5 and HYH bind to a T/G-box (CCACGTTC) necessary for *HY5* induction in response to UV-B. An E-box (CAATTGC), with less responsiveness to UV-B, was also necessary for *HY5* activation and represented the binding element for a HY5-interacting calmodulin (Abbas *et al.*, 2014). We screened the promoter regions (1kb upstream of the TSS) of all grapevine protein-coding genes for the presence of HY5 TFBS. We identified C/A-box, C-box, and C/G-box signatures in 41.1% (12 305 genes), 30.1% (9011 genes), and 35.0% (10 488 genes) of gene promoters, respectively. These frequencies closely match the proportion of total Arabidopsis promoters predicted to contain these motifs (~48%, 15 000 genes; Song *et al.*, 2008). A total of 35.8% (10 724 genes) and 21.1% (6333 genes) of promoters were predicted to contain an E-box and T/G-box, respectively. Positional bias analysis identified all motifs, except the E-box, to have a localization preference towards the TSS, as seen in both distribution plots and *Z*-score values (Fig. 4A). *Z*-scores ≥3 indicate a strong (and highly significant) position bias towards the TSS, thus supporting these elements as bona fide motifs (Ma *et al.*, 2013). The likelihood of random CRE occurrence and clustering towards the TSS was further evaluated in random promoters (1kb in length) simulated using the actual grapevine promoter base composition ratios (A:C:G:T=0.33:0.16:0.16:0.34) (Supplementary Fig. S7). This AT-rich ratio is similar to that of other plant promoter base compositions including Arabidopsis, rice, and soybean (Maruyama *et al.*, 2012). No positional bias was found towards the TSS (random motif occurrences along the promoter) and significantly fewer genes containing these CREs (*P*<0.01) were observed in random promoters, demonstrating the validity of the high proportion of grapevine promoters containing HY5/HYH- and UV-B-related CREs.

We determined the enrichment of these bona fide motifs in promoters of HY5/HYH CEGs, finding C/G-box motifs to be highly enriched (*P*<0.01) in most GCNs based on (i) proportions of genes containing the C/G-box (Fig. 4B; Supplementary Table S5) and (ii) the number of occurrences (Fig. 4C; Supplementary Table S6). To a lesser extent, the C-box and C/A-box were also enriched in HY5 stress-related RNA-seq GCNs (Fig. 4B). More than 40% of CEG promoters with at least 150 copies in each GCN contained a C/G-box. Together, these observations show that this motif is the predominant recognition site for VviHY5 and VviHYH based on GCNs. Indeed, RNA-seq coupled with ChIP-chip analysis revealed that C/G-box motifs were the most abundant (and highly enriched) within Arabidopsis HY5-regulated genes (Zhang *et al.*, 2011). Nonetheless, strong enrichment (*P*<0.01) of the C-box, C/A-box, and T/G-box (in addition to the C/G-box), especially in stress-related HY5 GCNs, provides support for a wider ACGT-core-binding spectrum of HY5 compared with HYH, where HYH might be specific for the C/G-box. Octamer scans derived from the consensus sequences reinforce a higher propensity of HYH to bind specifically to C/G-box motifs, while HY5 has a more diverse binding potential in promoters during development or under stress (Fig. 4D; Supplementary Table S7). This observation leads to a more diverse set of genes targeted by HY5 compared with HYH (for instance, AtHY5 can bind ~40% of the total coding genes, 11 000 genes; Zhang *et al.*, 2011).

**Fig. 5.**
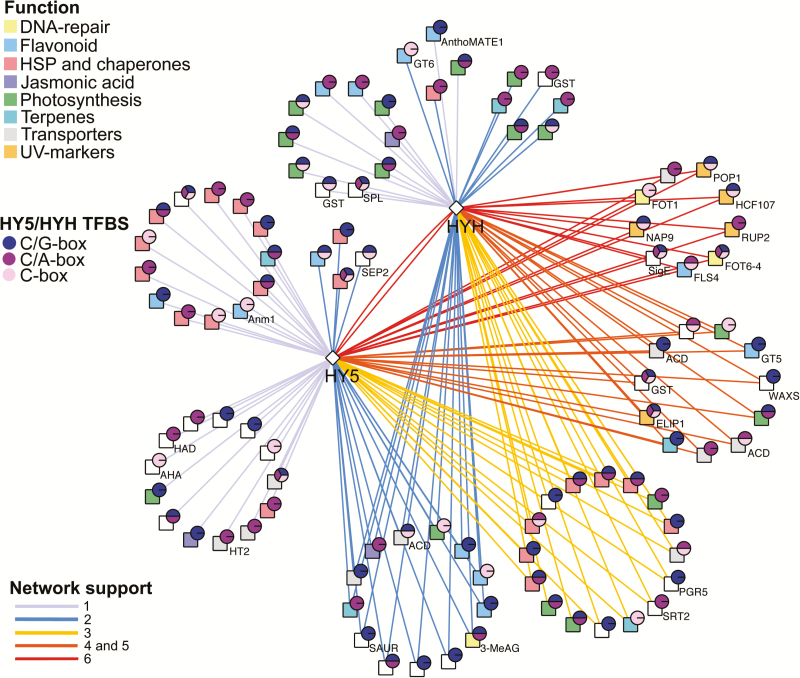
Integrated grapevine *HY5* and *HYH* community gene co-expression and *cis*-regulatory subnetwork. Square and circle nodes indicate co-expressed target genes and the presence of various HY5 consensus CREs predicted in co-expressed target genes. Square nodes indicate putative functions of co-expressed target genes; DNA repair, flavonoid, HSP and chaperones, jasmonic acid, photosynthesis, terpenes, transporters, and UV markers. Circle nodes, shown as pie charts, indicate the type of HY5 consensus CRE present in the promoter region of each co-expressed gene. Edges represent significant co-expression between each unique and shared HY5 and HYH subnetwork. Edges also indicate the nodes supported by one, two, three, four, five, and six co-expression networks. Gene IDs can be found in Supplementary Table S8. (This figure is available in colour at *JXB* online.)

To construct an integrated HY5 and HYH community network, we compared HY5/HYH networks with previous microarray and ChIP-chip analyses performed in AtHY5 for addressing AtHY5 regulatory networks in Arabidopsis. We constructed an overlapping network of VviHY5 and VviHYH high confidence targets (Fig. 5; Supplementary Table S8) based on different criteria: (i) co-expressed genes with network support; (ii) at least one HY5-CRE; and (iii) homology to an Arabidopsis gene differentially expressed in *hy5-1* mutants (Brown *et al.*, 2005) or that is a binding target of AtHY5 (Lee *et al.*, 2007; Zhang *et al.*, 2011).

HY5/HYH candidate targets represent good UV-B molecular markers for further gene expression studies. Among these, we found several orthologues of Arabidopsis light- and chloroplast-related genes (e.g. *HCF107*, *VIT_05s0077g01010*), ATP-binding cassette transporters (*NAP9*, *VIT_14s0060g00720* and *POP1*, *VIT_07s0005g03680*), RNA polymerases (e.g. *SigE*, *VIT_16s0050g02520*), and DNA repair enzymes (e.g. *FOT6-4*, *VIT_09s0002g05990*; *FOT1*, *VIT_02s0241g00040*, and *3-MeAG*, *VIT_01s0010g00020*). Photolyases form a family of flavoproteins (Supplementary Fig. S8) and are involved in both specific and non-specific UV-B signalling pathways. We show that these represent potential HY5/HYH targets. Finally, 18 heat shock-related genes (Class I and II heat shock proteins, chaperones, and transcription factors) were found in the specific-*HY5* stress data set (Fig. 5).

### The ectopic expression of HY5 validates its network associated with flavonol-related genes

We see a co-regulation of HY5/HYH with flavonol-related genes, being preserved in all our networks (Fig. 5; Supplementary Table S3). The most highly ranked case is *FLAVONOL SYNTHASE 4* (*FLS4*; Fujita *et al.*, 2006), also known as *FLS1* (Czemmel *et al.*, 2009), followed by *FLAVONOL-3-O-GLYCOSYLTRANSFERASE 5* (*GT5*, *VIT_11s0052g01600*). Flavonols are glycosylated in their last biosynthetic step, a process generally related to their posterior transport and accumulation in vacuoles. GT5 and its paralogue GT6 act as a UDP-glucuronic acid:flavonol-3-*O*-glucuronosyltransferase and a bifunctional UDP-glucose/UDP-galactose:flavonol-3-*O*-glucosyltransferase/galactosyltransferase, respectively (Ono *et al.*, 2010). Both GTs present several HY5 TFBS.

We previously demonstrated that the rapid light responsiveness of *FLS4* was mainly dependent on the grape R2R3-MYBF1 transcription factor, an orthologue of AtMYB12 (Czemmel *et al.*, 2009; Matus *et al.*, 2009). In Arabidopsis, flavonol synthesis is activated by a light- and UV-B-induced HY5-dependent mechanism, where AtHY5 binds to the AtMYB12 promoter (Stracke *et al.*, 2010). Although VviMYBF1 was not present in the top 300 co-expressed lists for HY5 and HYH, we found the presence of one C/G-box in the proximal region of its promoter (–129 bases from the TSS) and several moderate to low rankings (top 400–800) with modest correlations (PCC ~0.5) across the various data sets (data not shown). We thus tested the capacity of HY5 for regulating *MYBF1* together with *FLS4* and *GT5*.

**Fig 6.**
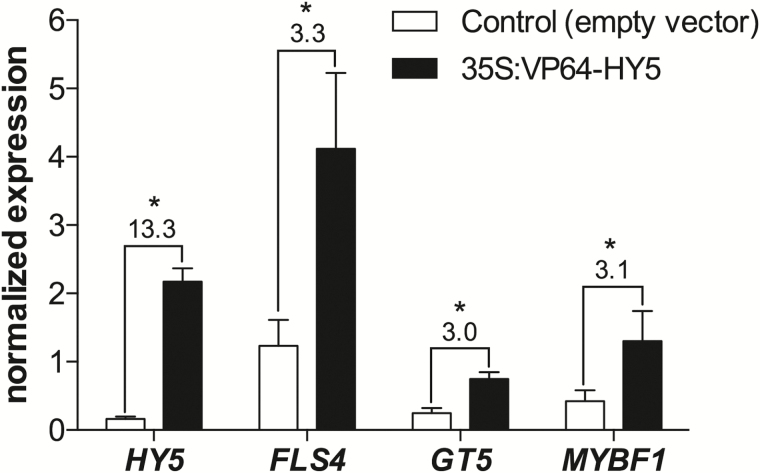
Transient expression of *HY5* in grapevine plantlets induces flavonol-related genes. Normalized gene expression values and induction fold changes in response to *HY5* ectopic expression. Grapevine *in vitro* plants were agroinfiltrated with either a 35S:VP64-HY5 construct or an empty vector, and kept in low light conditions for 5 d before gene expression quantification. Values above asterisks indicate significant differences compared with the control.

The H class of Arabidopsis bZIP transcription factors lacks a transcriptional activation domain. Expression of AtHY5 in yeast does not activate transcription itself and its overexpression has no effect on its targets genes (Ang *et al.*, 1998). However, the expression of an N-terminal fusion of the virion protein VP16 to AtHY5 led to the specific activation of a Pro*MYB12:GUS* reporter construct in darkness (Stracke *et al.*, 2010), revealing that HY5 requires co-operative partners for target activation. We designed a VP16×4(VP64)–VviHY5 construct which was later agroinfiltrated in grapevine *in vitro* plantlets. Plants were kept in low light conditions for 5 d to avoid high basal levels of the endogenous *HY5* caused by light induction. Plants with the highest expression of the transgene also had increased levels of *MYBF1* and *GT5*, and a very high induction of *FLS4* (Fig. 6). Our results identify two crucial steps of flavonol accumulation (synthesis and modification) to be targeted by HY5, and possibly HYH. Additionally, a higher hierarchy of regulation is also present (i.e. regulation of regulators), thus inducing a direct and indirect activation of *FLS4* and additional targets.

### UV-B radiation in leaves modifies the expression of *HY5* and *HYH* and a set of CEGs including genes related to the synthesis of flavonols

**Fig. 7.**
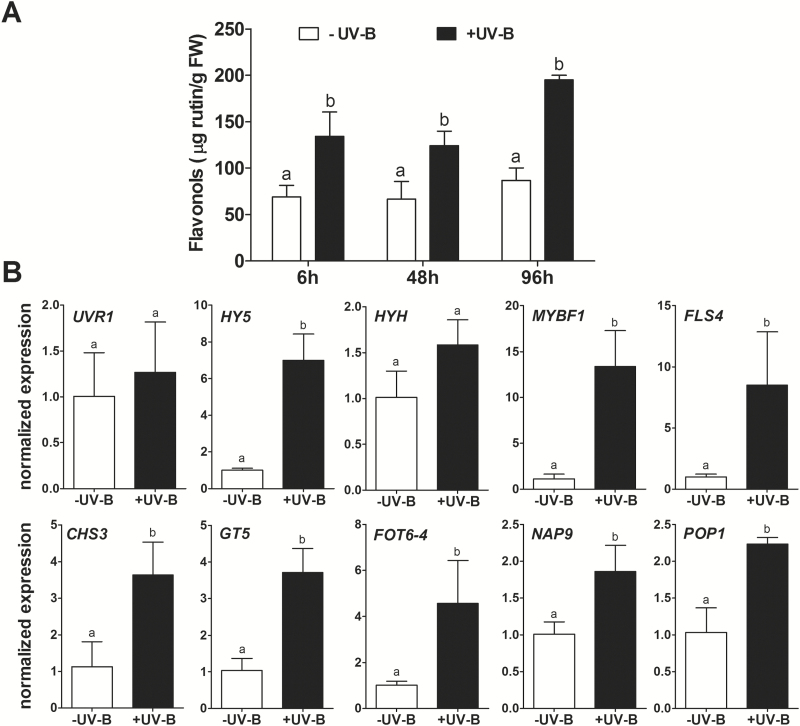
Flavonol synthesis is activated in leaves in response to low UV-B irradiance. (A) Time series of flavonol accumulation in plantlets exposed to 6h of UV-B under *in vitro* conditions. The 48h and 96h measurements correspond to the recovery stage after the treatment. Data were normalized against the control (–UV-B), independently for each time point. Different lower case letters indicate significant differences between treatments as calculated by Tukey statistical analysis (*P*<0.05). (B) Expression of a set of *VvHY5* co-expressed genes in plantlet leaves after 6h of irradiation with low UV-B irradiance. Gene expression was measured by quantitative real-time PCR, and data were normalized against the control.

We examined the responsiveness of putative UV-B marker genes in vegetative grapevine organs. As described in Cavallini *et al.* (2015), we exposed *in vitro* plantlets to UV-B radiation for 6h (irradiance of 0.15W m^-2^, Supplementary Fig. S9). We observed an increase in the flavonol content (Fig. 7A) and in the expression of regulatory and structural genes related to flavonoid synthesis (Fig. 7B). This up-regulation correlated to the expression of *HY5* but not to the expression of *UVR1*. We also observed an up-regulation of the highly confident HY5 targets, *FOT6-4*, *NAP9*, and *POP1*. AtHY5 activates the expression of *AtCHS* and *AtFLS*, increasing flavonol glycoside levels under UV-B (Oravecz *et al.*, 2006; Favory *et al.*, 2009; Stracke *et al.*, 2010). In our experiment, the accumulation of total flavonols was significantly higher at all time points (6, 48, and 96h following the start of the 6h treatment). *MYBF1*, *FLS4*, and *GT5*, all HY5 targets, were significantly induced at 6h (Fig. 7B). Their promoters present several ACEs, which in Arabidopsis were demonstrated by Stracke *et al.* (2010) and Shin *et al.* (2013) to be bound by AtHY5 and essential for the responsiveness of *AtMYB12* and *AtMYB75*/*PAP1* to light or UV-B.

To determine if *HYH* was induced at other time points in response to UV in leaves, we performed a time-series experiment by irradiating leaves from grapevine stem cuttings with UV-B and UV-A. As seen in Supplementary Fig. S10, the expression of *HY5*, *HYH*, *FLS4*, and *MYBF1* was rapidly induced at 10h after the treatment. *FLS4* expression peaked at 24h, after the highest expression of *MYBF1*, *HY5*, and *HYH*, but also showed a second peak at 72h in concordance with a subsequent induction of *MYBF1* and *HYH*. Altogether, these results show that HY5 and HYH are UV responsive in vegetative organs, but differ in both the timing and magnitude of their response.

### Enhanced levels of flavonols correlate with an up-regulation of *HY5* and *HYH* in fruits

**Fig. 8.**
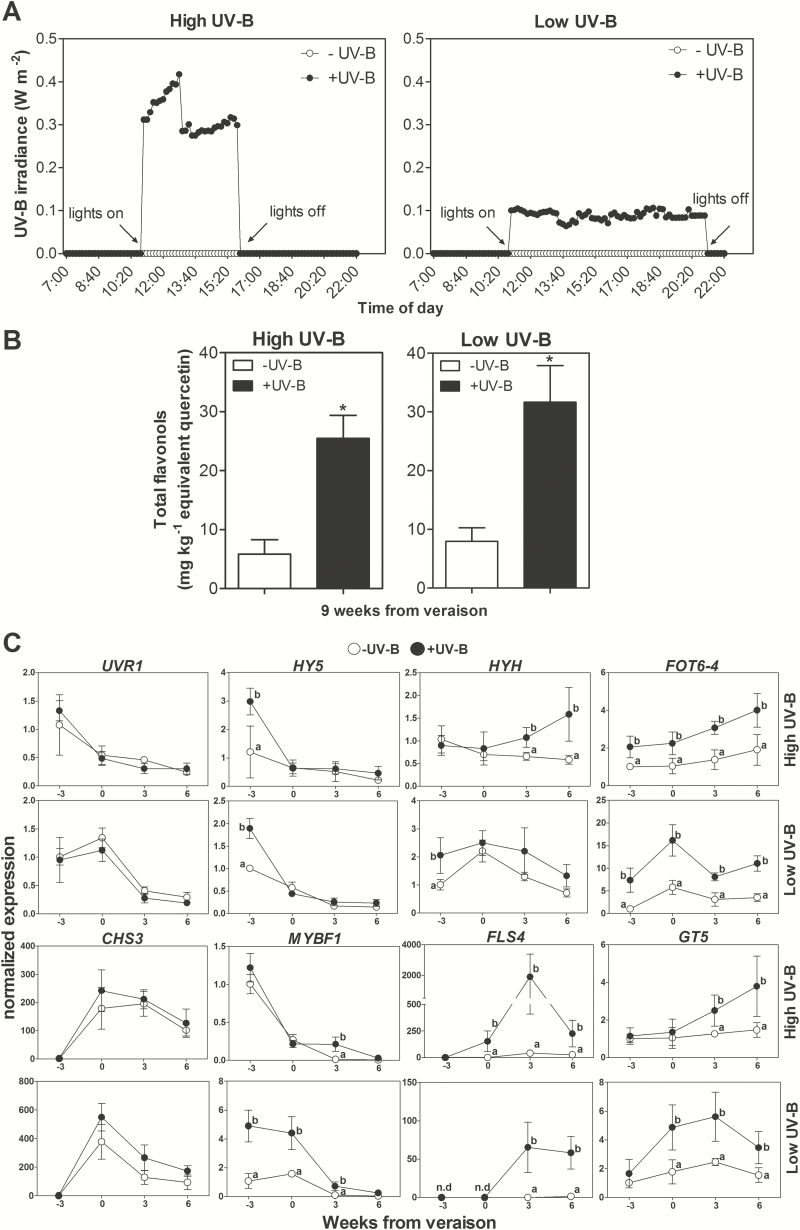
The UV-induced expression of flavonol-related genes correlates with *HY5* and *HYH* at different stages of berry development. (A) UV-B daily measurements were taken in a tested UV-free greenhouse from 07:00h to 22:00h. (B) Flavonol measurements at technical maturity in berry skins in response to high and low UV-B irradiance at 9 WAV. (C) Gene expression changes of the UV-B signalling pathway and flavonoid-related genes in berry skins of irradiated fruit clusters. Gene expression data were normalized against the control at –3 WAV. Different lower case letters indicate significant differences (Tukey test, *P*<0.05).

To determine if flavonol synthesis was also activated and correlated with *HY5* and *HYH* in reproductive organs, we performed an irradiation experiment on fruits of 15-year-old potted plants growing in a phytotron under controlled environmental conditions. Low (0.1W m^−2^) and high (0.3W m^−2^) UV-B irradiance assays were performed in the same plants during different seasons (with their respective controls). UV-B lamps were kept at two different distances from the grape clusters and irradiated photo-periodically from fruit set onwards (long-term experiment classification according to Martínez-Luscher *et al.*, 2013). The same daily biologically effective exposure was used for both treatments by applying different times of exposure (Fig. 8A). Between one season and the other, similar and moderate environmental fluctuations were observed (Supplementary Fig. S11); therefore, any difference in gene expression could be mostly attributed to differences in the applied UV-B. Figure 8B shows that the total flavonol content at technical maturity (9 WAV) was significantly higher upon high or low UV-B exposure (Supplementary Fig. S12).

General tendencies in gene expression profiles for control conditions (–UV-B) were maintained for all genes in the two experimental seasons (Fig. 8C). Only *UVR1* and *MYBF1* showed a slight difference between seasons; their expression dropped more intensely at veraison in season 2011–2012 while this down-regulation was delayed by ~3 weeks in season 2012–2013. *UVR1* gene expression was unaffected by low or high radiation in the same manner as observed in low fluence-irradiated plantlets. On the other hand, *HY5* was induced in response to low and high UV-B irradiance in green-stage berry skins. *HYH* expression increased in high fluence UV-B at ripening, and more strikingly by the low radiant exposure treatment before veraison (Fig. 8C).

UV-B marker genes such as the photolyase *FOT6-4* were induced at all time points in both types of irradiances, showing that its transcriptional regulation is crucial in HY5/HYH-specific UV-B responses. *MYBF1* expression decreased at mid-point stages of ripening, described previously by Czemmel *et al.* (2009). However, it showed a slight up-regulation with the high fluence treatment at 3 WAV and maintained induction from green to ripe berry skins with low UV-B. Both *FLS4* and *GT5* showed an increase in expression throughout development, with a major up-regulation upon both UV-B exposures. In the case of *FLS4* at high UV-B levels, it peaked at 3 WAV, while for the low exposure this increase was kept constant between 3 and 6 WAV. *GT5* showed different expression between both types of radiation, reflecting an earlier up-regulation by low UV-B. These results support the idea that HY5, HYH, and MYBF1 regulate *FLS4*; as suggested by our *in silico* analysis.

### UV-B response factors are modulated by light, temperature, and biotic stress pathways

**Fig. 9.**
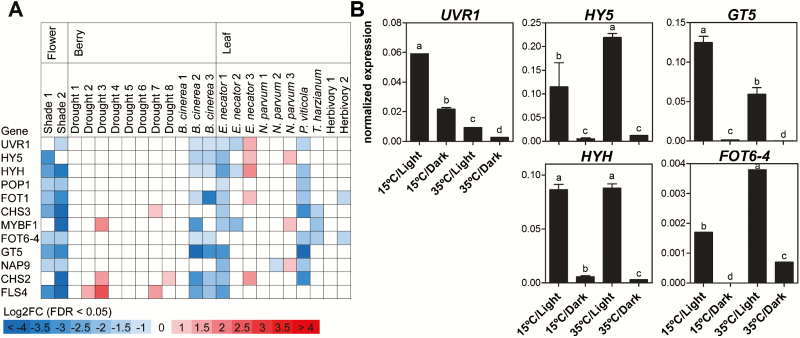
UV-B response factors are modulated by light, temperature, and biotic stress. (A) Expression analysis of candidate UV-B perception and signalling in response to various abiotic and biotic stresses in different organs based on RNA-seq data. Significantly up-regulated and down-regulated (FDR <0.05, |Log2FC|>1) genes identified with DESeq2 are indicated with varying intensities. (B) Expression of UV-B response factors under both light and temperature regimes in detached grape berries. Different lower case letters indicate statistically significant differences (*P*<0.05). (This figure is available in colour at *JXB* online.)

We searched for additional regulatory scenarios of these photomorphogenic factors. Re-analysis of RNA-seq stress data sets showed severe down-regulation of *UVR1*, *HY5*, and *HYH* upon shading, but, unexpectedly, these were also biotic stress responsive (Fig. 9A). These genes were down-regulated in co-ordination with their targets in *Botrytis cinerea*-infected berries (noble rot) and *Erysiphe necator*- (powdery mildew) or *Plasmopara viticola*- (downy mildew) infected leaves. This observation has not been reported before for any other HY5 homologue. Down-regulation of these genes could be explained by the fact that biotic stress represses several photosynthesis and light-related genes across the plant kingdom, regardless of the vector causing the damage (Bilgin *et al.*, 2010). Thus it could be possible that the fungus machinery is capable of directly repressing the role of HY5. The only case of up-regulation was observed in leaves during latent stages of *E. necator* (and *Neofusicoccum parvum*) infection.

To confirm the expression data in response to shade, we evaluated the expression of the UV-B signalling factors in an *in vitro* experimental approach defined by Azuma *et al.* (2012), using detached grape berries cultured under different light and temperature conditions. *UVR1* expression was dramatically down-regulated in the dark, with high temperature also playing a repressive role (Fig. 9B). The expression levels of *HY5* and *HYH* were prominently affected by light exclusion, while high temperature only had a positive influence on *HY5* expression (*HY5* expression data were previously shown by Azuma *et al.*, 2015). This finding gives support to the role of HY5 regulating a large set of heat shock proteins (Fig. 5). Additionally, *GT5* expression was reduced in dark conditions in a strong interaction with high temperature. Conversely, *FOT6-4* expression was up-regulated by high temperature and dramatically reduced in the dark, similarly to *HY5*.

### Final remarks

We provide a wide overview of gene function and expression behaviour of UV response genes in the grapevine. The complementation assays of *uvr8* and *hy5* mutants demonstrate that both proteins conserve their role in the UV-B signalling pathway. We determine a high confidence set of HY5 and HYH targets. The targeted *in planta* assay shows the capacity of HY5 to induce the expression of flavonol-related genes in grape and demonstrates the robustness of combining promoter and gene co-expression bioinformatic studies.

Our results show conserved roles for the grapevine UV-B response factors compared with Arabidopsis. However, they diverge at the expression and regulatory level. Organ-related expression of the UV-B receptor is dissimilar between both species as *AtUVR8* is ubiquitously expressed while *UVR1* is strongly induced in early berry developmental stages. *VviUVR1* expression was not affected by UV-B itself (like in Arabidopsis) but it was affected by light exclusion and temperature, in contrast to AtUVR8 levels that seem unresponsive to different light qualities (Kaiserli and Jenkins, 2007). This differential regulation between the two UV-B receptors reflects different adaptation strategies to solar radiation, especially regarding the initial steps of fruit development. However, and even though *UVR1* expression levels were reduced after veraison, we cannot discard a basal UV-B perception mechanism still present in ripening berries due to maintained protein levels of this receptor.

The expression of *HY5* and *HYH* has clearly subspecialized. *HY5* expression coincides with that of *UVR1* at early berry developmental stages, when this organ is more sensitive to solar radiation due to the absence of sun-screening compounds (e.g. anthocyanins). In contrast, HYH has a secondary role that becomes predominant at ripening. This diversification could represent a grapevine-specific feature, as in other species such as Arabidopsis, HY5 and HYH are more ubiquitously expressed throughout the plant. These differences suggest (i) additional mechanisms regulating *HYH* expression and (ii) that HY5 and HYH roles may complement each other in a time-, organ-, and possibly stimulus-dependent manner, a hypothesis that is strengthened when comparing berry developmental stages.

Our results imply overlapping roles for HY5 and HYH. First, both respond at different times in irradiated leaves, where HYH could have a secondary role (although maintained in time), in contrast to the strong, fast, and short response of HY5. This overlap is also demonstrated at the level of GCNs presented here. The Arabidopsis homologues also share partially overlapping functions in light-dependent development (Holm *et al.*, 2002). Despite our results showing a major response to UV-B radiation, it is difficult to study the effects of UV-B without exposure to UV-A. By filtering UV-B, we have filtered the direct effects of UV-B but also the indirect effects of UV-B modifying UV-A responses. Further studies should include the use of narrower band lamps to assess specific effects of UV-B and UV-A.

UV-B perception and signalling orchestrates the strong accumulation of flavonols in grape reproductive and vegetative organs, by inducing structural and regulatory genes of the flavonol branch within the phenylpropanoid pathway. This mechanism is vastly conserved in the plant kingdom. However, the long-term UV-B adaptive mechanisms that grapevines possess are highly efficient in part due to the activation of HY5 and HYH in response to high UV-B, in addition to their conserved photomorphogenic response to low levels of radiation. This is in contrast to what occurs in Arabidopsis, where different types of UV-B levels independently activate specific or non-specific signalling pathways. Grapevines also synthesize additional secondary metabolites that may improve their fitness under increasing radiation environments. Such is the case of carotenoid and terpenoid compounds that allow a rapid UV-B acclimation depending on the light qualities influenced by canopy management (Joubert *et al.*, 2016; Young *et al.*, 2016). These changes in secondary metabolism, in addition to other physiological responses (e.g. increase of photosynthetic rates, changes in source-to-sink carbon fluxes, etc.), situate *Vitis* species as a model system to evaluate adaptive responses to radiation.

## Supplementary data

Supplementary data are available at *JXB* online

**Figure S1.** Phylogenetic analysis of VviUVR1- and UVR8-related proteins.

**Figure S2.** Phylogenetic relationships between HY5 homologues.

**Figure S3.** RT-PCR detection of the *VviUVR1* and *VviHY5* transgenes in Arabidopsis mutants.

**Figure S4.** Hypocotyl length and flavonol accumulation in *uvr8-6* and *hy5-215* complemented lines under dark conditions.

**Figure S5.** Expression profiles of *UVR1*, *HY5*, and *HYH* in grapevine organs of cv. Corvina.

**Figure S6.** Summary of enriched Gene Ontology (GO) biological process (BP) terms from *HY5* and *HYH* gene co-expression networks.

**Figure S7.** Genome-wide analysis of HY5 *cis*-regulatory elements in random promoter sequences.

**Figure S8.** Relationships between grapevine photolyases and cryptochrome-related flavoproteins.

**Figure S9.** Experimental set-up of *in vitro* plantlets exposed to low UV-B irradiance.

**Figure S10.** Up-regulation of *HY5*, *HYH*, and the flavonol-related *MYBF1* and *FLS4* genes in UV+light-treated grapevine stem cuttings.

**Figure S11.** Environmental parameters measured during the UV-B fruit exposure experiments.

**Figure S12.** HPLC chromatograms of flavonol analysis for the low UV-B treatment at 9 WAV.

**Table S1.** List of primers used in this study.

**Table S2.** Summary of RNA-seq data sets and associated meta-data used in the construction of the stress-related gene co-expression network in this study.

**Table S3.** Summary of grapevine *HY5* and *HYH* co-expressed genes aggregated from three different gene co-expression networks.

**Table S4.** Summary of enriched Gene Ontology terms in the grapevine *HY5* and *HYH* co-expressed gene network constructed from the atlas (this study), stress-related RNA-seq (this study), and stress-related VTCdb data set.

**Table S5.** Summary of enriched *HY5*- and UV-related CREs in the promoter region (1kb upstream of the TSS) of co-expressed genes in the various *HY5* and *HYH* co-expressed gene networks.

**Table S6.** Summary of CRE occurrences in promoters of co-expressed genes in various *HY5* and *HYH* gene co-expression networks.

**Table S7.** Summary of enriched individual octamer combinations of HY5 consensus CREs in the promoter region (1kb upstream of the TSS) of co-expressed genes in the various *HY5* and *HYH* co-expressed gene networks.

**Table S8.** Genes used for the HY5/HYH co-expression community and *cis*-regulatory subnetwork.

## Supplementary Material

Supplementary Table

Supplementary Figures

## Abbreviations:

CEG: co-expressed gene
CRE: *cis*-regulatory element
GCN: gene co-expression network
