# Supplementary material for: The photomorphogenic factors UV-B RECEPTOR 1, ELONGATED HYPOCOTYL 5, and HY5 HOMOLOGUE are part of the UV-B signalling pathway in grapevine and mediate flavonol accumulation in response to the environment
Source: J Exp Bot. 2016 Aug 19;67(18):5429–45. doi: 10.1093/jxb/erw307 (PMC5049392; doi:10.1093/jxb/erw307)
Supplement: Supplementary Figures [file erw307_suppl_supplementary_figures_s1_12.pdf]

Supplementary Figures

Figure S1 (next page)

Phylogenetic analysis of VviUVR1 and UVR8-related proteins from different plant species. (A) Full-length protein sequence alignment of *Vitis vinifera* UVR1 and its closest homologs from *Arabidopsis thaliana*, *Thellungiella halophile*, *Populus trichocarpa*, *Ricinus communis* and *Physcomitrella patens*. Identical residues are shown in black and conserved residues in dark gray. Regulator of chromosome condensation (RCC1) domains are shown. Tryptophan (W) residues are indicated by asterisks. (B) Neighbor-joining phylogenetic tree of plant UV-B receptor proteins, using UVR8-like proteins from *P. trichocarpa*, *R. communis*, *Glycine max*, *A. thaliana*, *T. halophile*, *P. patens*, *Oryza sativa* and *Brassica rapa*. *A. thaliana* PHYA and PHYB proteins were used as out-groups. Bootstrap values are indicated at each branch. The scale bar represents 0.1 substitutions per site. GenBank accession numbers: VviUVR1 (AGG82479); AtUVR8 (Q9FN03); ThUVR8 (BAJ33982); PtUVR8 (EEE90389); RcUVR8 (EEF39472); PpUVR8 (EDQ78262); BrUVR8 (ABV89658); GmUVR8 (XP_003526878); OsUVR8 (ABA94217); AtPHYA (AAA21351); AtPHYB (AAR32737). Sequence alignments were performed using MEGA5 software (**Tamura *et al.*, 2011**) and the MUSCLE algorithm (**Edgar, 2004**). Phylogenetic trees were constructed with the Neighbor-Joining method in MEGA5. The tree nodes were assessed by bootstrap values generated from 2000 retrials.

A

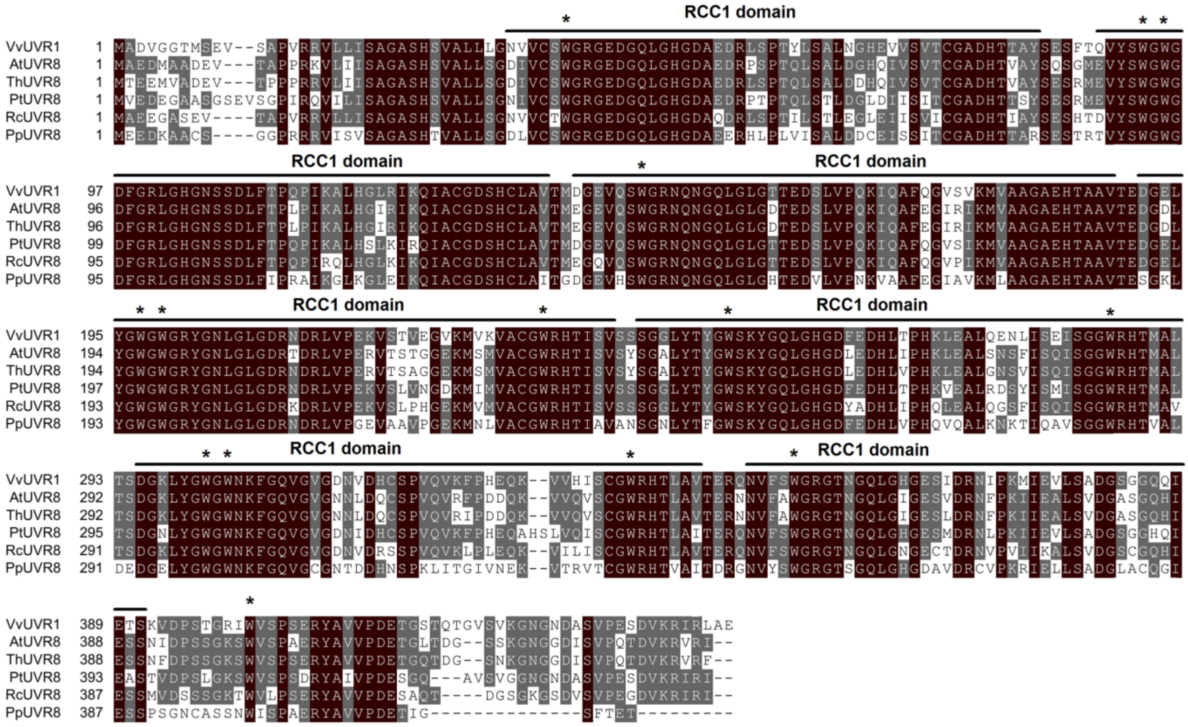
RCC1 domain
VvUVR1 1
AtUVR8 1
ThUVR8 1
PtUVR8 1
RcUVR8 1
PpUVR8 1
VvUVR1 97
AtUVR8 96
ThUVR8 96
PtUVR8 99
RcUVR8 95
PpUVR8 95
VvUVR1 195
AtUVR8 194
ThUVR8 194
PtUVR8 197
RcUVR8 193
PpUVR8 193
VvUVR1 293
AtUVR8 292
ThUVR8 292
PtUVR8 295
RcUVR8 291
PpUVR8 291
VvUVR1 389
AtUVR8 388
ThUVR8 388
PtUVR8 393
RcUVR8 387
PpUVR8 387

B

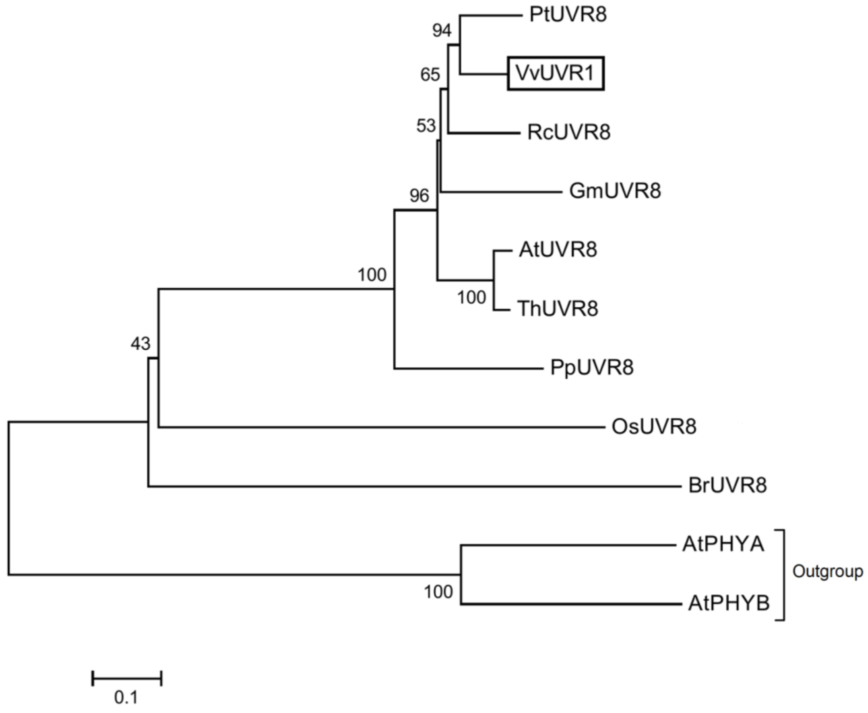
PtUVR8
VvUVR1
RcUVR8
GmUVR8
AtUVR8
ThUVR8
PpUVR8
OsUVR8
BrUVR8
AtPHYA
AtPHYB
Outgroup
94
65
53
96
100
100
43
100
0.1

Figure S2 (next page)

Phylogenetic relationships between VviHY5, VviHYH and their homologs. (A) Full-length protein sequence alignment with the closest homologs from *A. thaliana*, *Malus domestica*, *B. rapa*, *Solanum lycopersicum*, *Morus notabilis*, *R. communis* and *P. trichocarpa*. VviHY5 and VviHYH share 36% identity. The two grape proteins possess the conserved bZIP domain, which consists of a basic region mediating sequence-specific DNA-binding followed by a leucine zipper region required for dimerization. Identical residues are shown in black and conserved residues in dark gray. The COP1 interaction site, basic region and leucine zipper region are shown. The COP1-interaction site within motif 1 is preceded by an *in vivo* functional casein kinase II (CKII) phosphorylation site (**Oyama *et al.*, 1997; Hardtke *et al.*, 2000).** This sequence is formed by a cluster of 4-5 polar amino acids and is highly conserved in HY5 and HYH relatives. In Arabidopsis, these two sites are required for the physical interaction between AtHY5 and AtCOP1 (**Holm *et al.*, 2001**), an interaction that negatively regulates AtHY5 activity in the absence of light (**Osterlund *et al.*, 2000**). (B) Phylogenetic tree of different HY5 and HYH proteins from *A. thaliana*, *M. domestica*, *B. rapa*, *S. lycopersicum*, *M. notabilis*, *R. communis*, *P. trichocarpa*, *G. max*, *Pisum sativum*, *O. sativa*, *Z. mays* and *V. vinifera*. RUP1 and RUP2 proteins from *A. thaliana* were used as out-groups. Neighbor-joining tree with bootstrap values out of 2000 retrials. Scale bar represents 0.1 substitutions per site. GenBank accession numbers are: VviHY5 (AGX85877); VviHYH (AHX24181); AtHY5 (BAA21327); AtHYH (NP_850605); MdbZIP (BAM71071); BrHY5 (ABY83460); MnHY5 (EXC71566); SlHY5 (NP_001234820); RcHY5 (EEF43212); PtHY5 (EEF00511); GmSTF1 (AAC05017); PsLONG1 (ACP28171); OsHY5 (BAD35451); ZmHY5 (NP_001147637); AtRUP1 (Q9LTJ6); AtRUP2 (Q9FFA7).

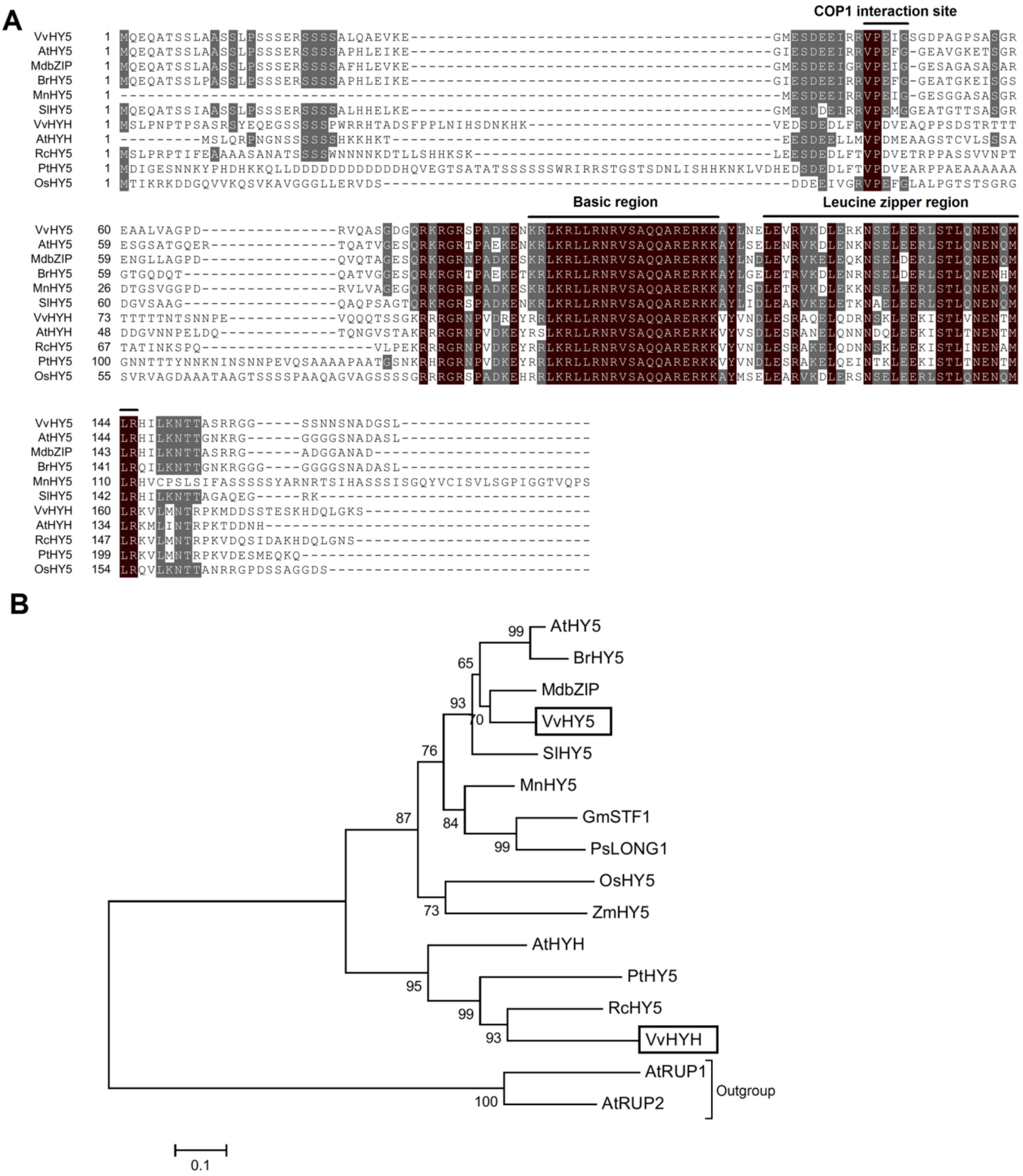
A
COP1 interaction site
Basic region
Leucine zipper region
VvHY5
AtHY5
MdbZIP
BrHY5
MnHY5
SlHY5
VvHYH
AtHYH
RcHY5
PtHY5
OsHY5
B
AtHY5
BrHY5
MdbZIP
VvHY5
SlHY5
MnHY5
GmSTF1
PsLONG1
OsHY5
ZmHY5
AtHYH
PtHY5
RcHY5
VvHYH
AtRUP1
AtRUP2
Outgroup
0.1

Figure S3

RT-PCR detection of the *VviUVR1* (A) and *VviHY5* (B) transgenes in Arabidopsis *uvr8-6* and *hy5-215* complemented lines, respectively. *AtACTIN2* was used as a control for cDNA integrity. Wt: wild type Col-0 plants. #: number of independent transgenic line.

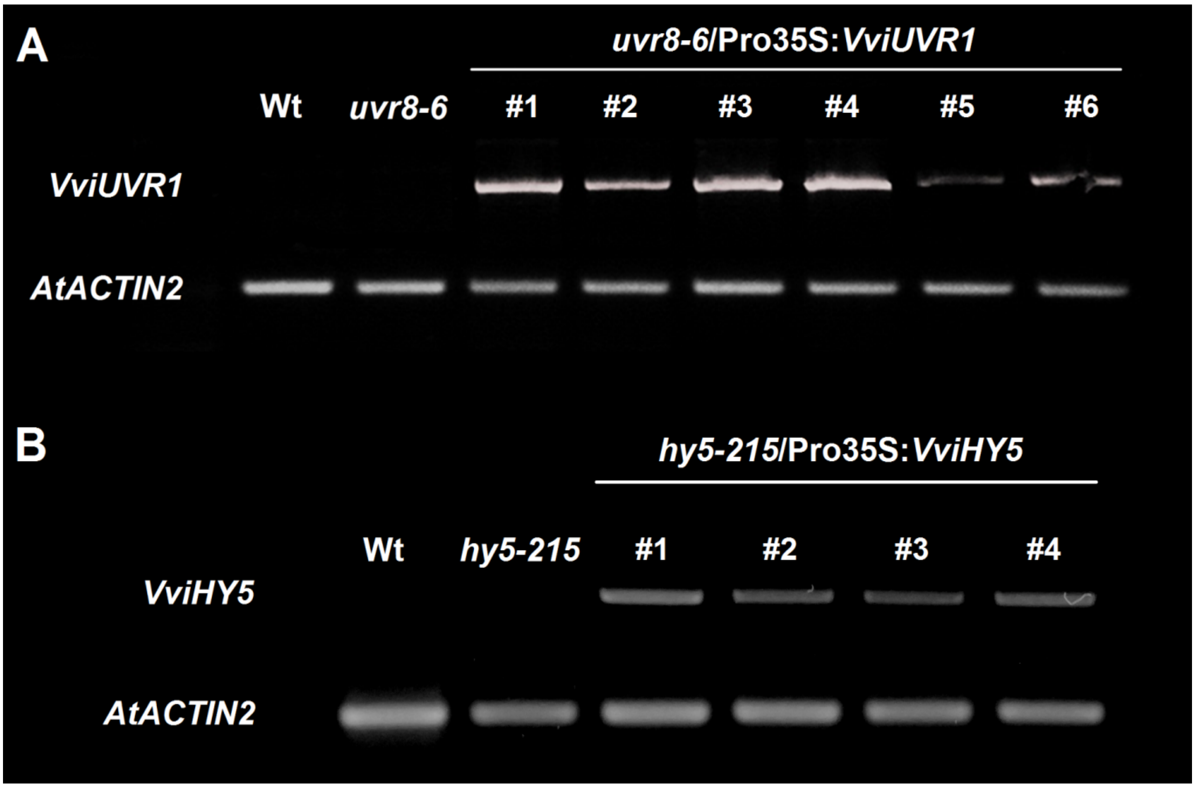

Figure S4

Hypocotyl length and flavonol accumulation in *uvr8-6* and *hy5-215* complemented lines under dark conditions. (A-D) Hypocotyl length in Wt (Col-0), *uvr8-6* mutant, *hy5-215* mutant, and *UVR1* or *HY5* complemented lines. (E-F) Total flavonol accumulation in six-day-old seedlings grown under continuous dark. Error bars represent the standard deviation (SD, n=6 plates with 15 seedlings each). Statistical analysis didn't show any difference between genotypes.

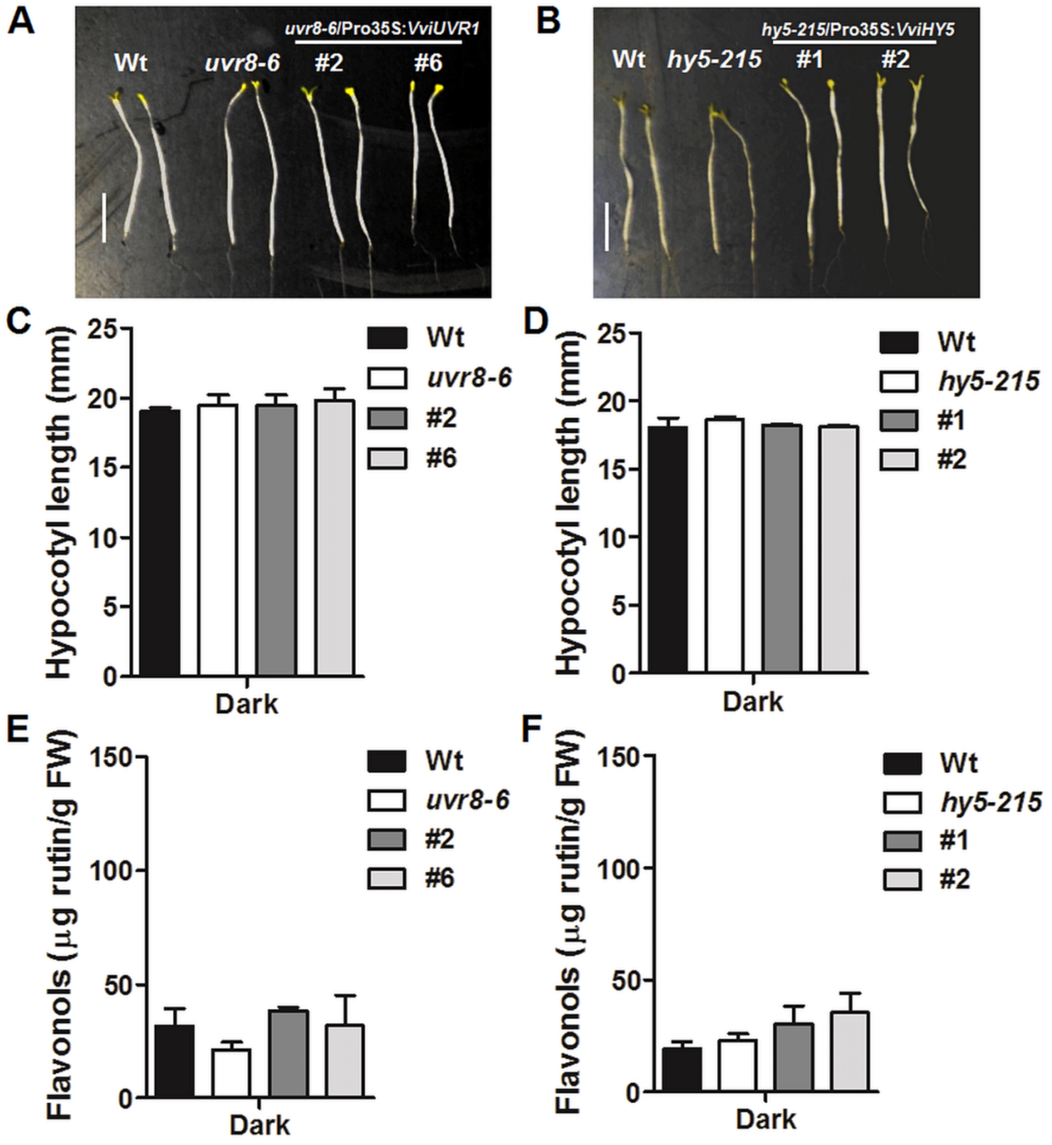

Figure S5

Expression profiles of *UVR1*, *HY5* and *HYH* in grapevine organs of cv. Corvina. The expression profiles of *UVR1*, *HY5* and *HYH* were assessed in the global *V. vinifera* cv. Corvina (clone 48) gene expression atlas (Nimblegen platform) of different organs at various developmental stages. The fluorescence intensity values of each transcript in all organs were calculated as log2 and normalized, based on the mean expression value of each gene in all organs. The colour scale bar represents expression levels with respect to the mean value. Abbreviations after organ names indicate the developmental stage. FS, fruit set; PFS, post fruit set; V, veraison; MR, mid-ripening; R, ripening; PHWI, post-harvest withering (1st month); PHWII, post-harvest withering (2nd month); PHWIII, post-harvest withering (3rd month), Bud - L, latent bud; Bud - W, winter bud; Bud - S, bud swell; Bud - B, bud burst; Bud - AB, bud after burst; Inflorescence - Y, young inflorescence with single flowers separated; Inflorescence - WD, well developed inflorescence; Flower - FB, flowering begins; Flower - F, flowering; Tendril - Y, young tendril; Tendril - WD, well developed tendril; Tendril - FS, mature tendril; Leaf - Y, young leaf; Leaf - FS, mature leaf; Leaf - S, senescing leaf; Stem - G, green stem; Stem - W, woody stem.

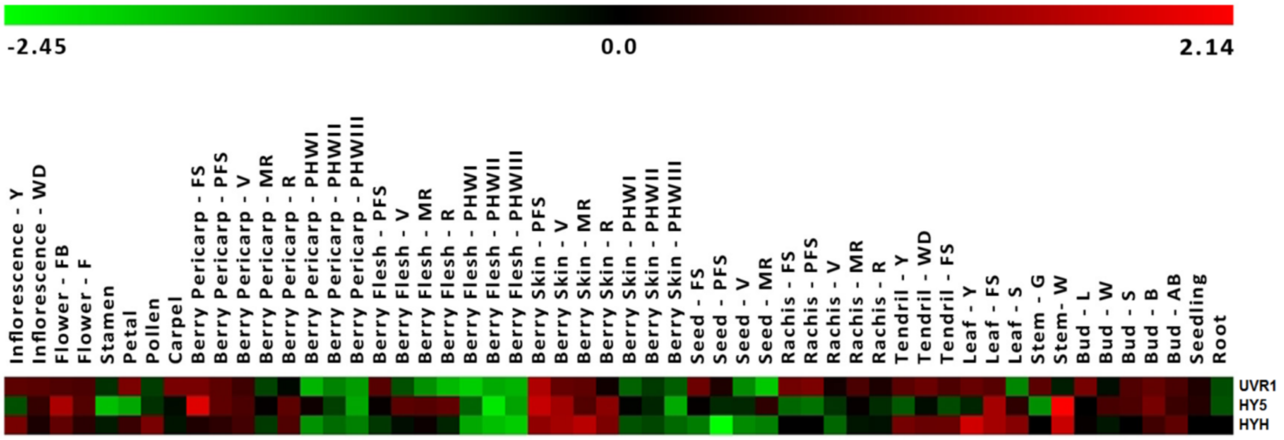

Figure S6

Summary of enriched gene ontology (GO) biological process (BP) terms from *HY5* and *HYH* gene co-expression networks. Enrichments, expressed as –log10(FDR) for each category are represented in a heatmap of colours in red (with varying intensities) and white, representing enriched and non-enriched categories for each GCN.

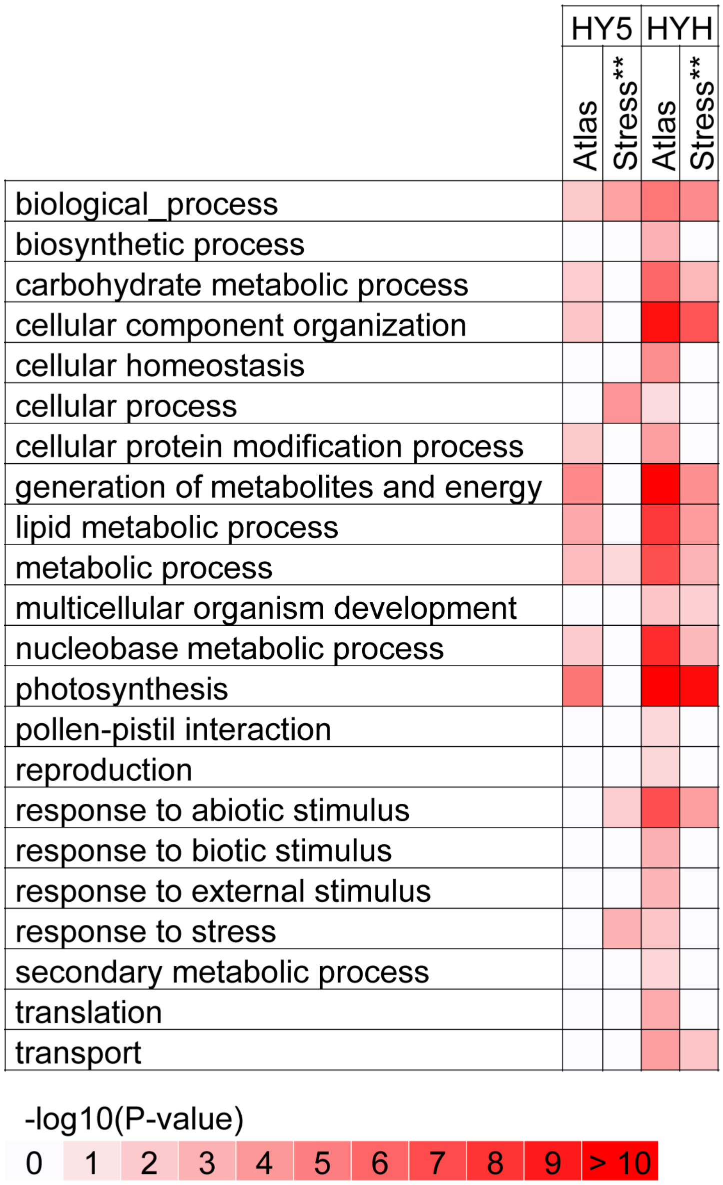

Figure S7

Genome-wide analysis of predicted HY5 and UV-B response cis-regulatory elements (CRE) in 29,839 random promoters sequences. (A) Distribution plots of HY5 and UV-B response CRE in random simulated promoter sequences. Each bin represents the total motif occurrence in 50 promoter bases, adjusted for the baseline occurrence. The baselines of C-box, C/A-box, C/G-box, E-box, and T/G-box in random simulated promoters are 316, 646, 313, 482, and 238, respectively. (B) Fold-enrichment of grapevine genes containing the CRE (Obs) compared to those identified in random simulated promoters (Exp) based on averages of 100 permutation observations.

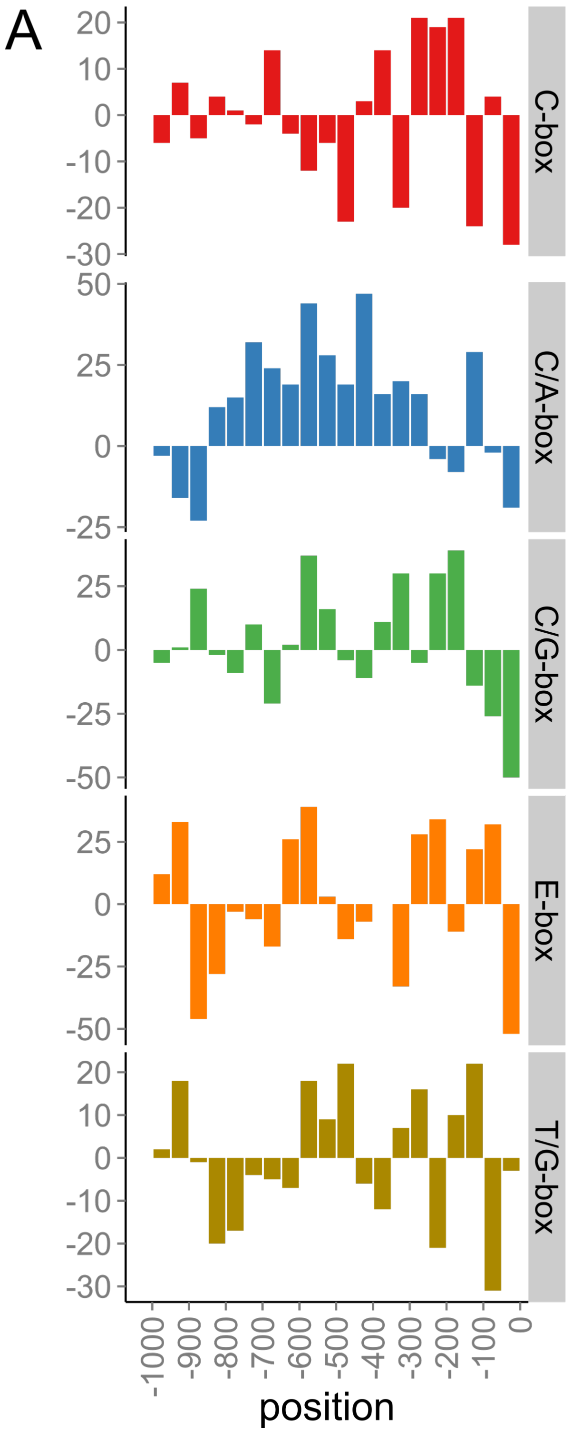

B

	Obs	Exp(Avg)	Obs/Exp
C-box	9011	5710	1.58
C/A-box	12305	10579	1.16
C/G-box	10488	5700	1.84
E-box	10724	8281	1.30
T/G-box	6333	4376	1.45

Figure S8

Relationships between grapevine photolyases and cryptochrome related flavoproteins. (A) The light-harvesting cofactor binding domain (cdd: pfam00875) was used in a search for all putative cryptochrome and photolyase flavoproteins in the grapevine 12Xv1 genome prediction. (B) Phylogenetic relationships between Arabidopsis and grapevine CRY-like flavoproteins. The blue-light photoreceptors PHOTOTROPIN 1 and 2 were used as outgroups.

A

Gene	Gene model 12X V1	Identities	Id %	Exp	Description
CRY1	VIT_18s0001g05680	74/166 (160)	44	8e-30,	Cryptochrome 1
CRY2	VIT_05s0049g00960	64/166 (160)	38	1e-25,	Cryptochrome 2
FOT6-4	VIT_09s0002g05990	63/147 (160)	42	2e-24,	6-4 photolyase (UVR3-like)
FOT2	VIT_07s0005g00590	52/128 (160)	40	6e-22,	Deoxyribodipyrimidine photolyase
CRYD	VIT_04s0008g02670	47/120 (160)	39	8e-17,	Cryptochrome DASH
FOT3	VIT_16s0022g01340	37/115 (160)	32	5e-11,	Deoxyribodipyrimidine photolyase
FOT1	VIT_02s0241g00040	44/138 (160)	31	3e-05,	Cyclobutane pyrimidine dimer photolyase (PHR1/UVR2-like)

B

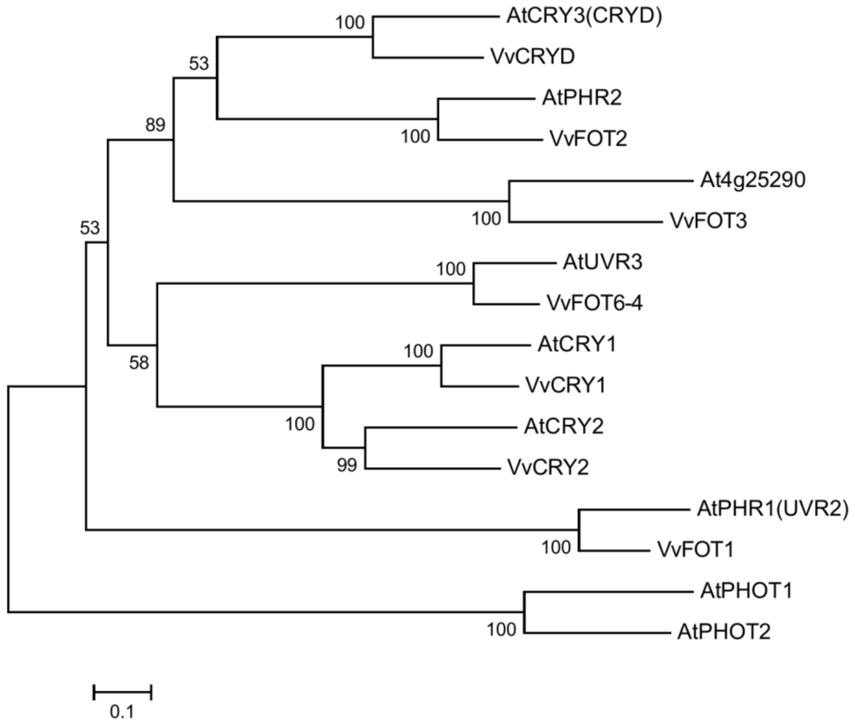

Figure S9

Experimental setup of *in-vitro* plantlets exposed to low fluence UV-B radiation (Cavallini *et al.*, 2015). (A) Tubes were covered with cellulose acetate filters that do not remove any UV-B radiation but exclude wavelengths lower than 280 nm. Percentage of PAR (B) and UV-B radiation (C) absorbed by 100 µm clear polyester film (PE film) (gray) used in -UV-B treatment.

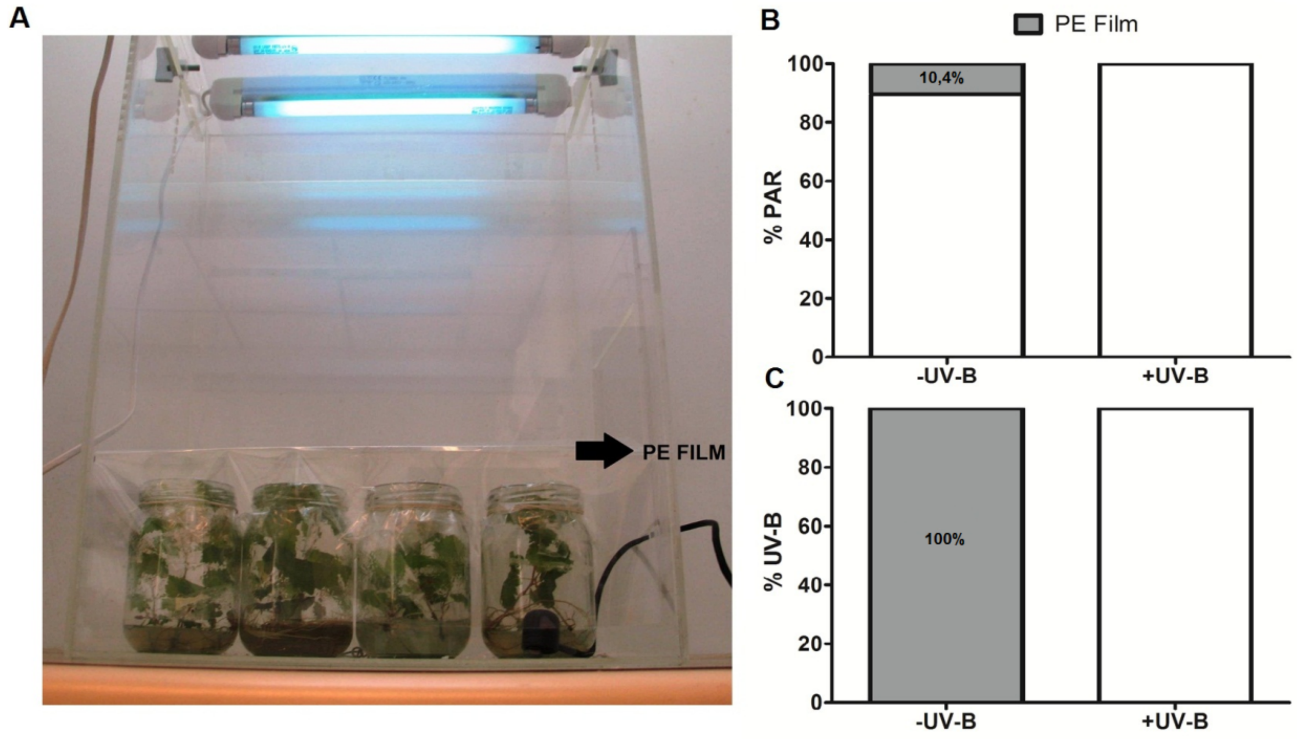

Figure S10

Up-regulation of *HY5*, *HYH* and the flavonol related *MYBF1* and *FLS4* genes in UV-B treated grapevine stem cuttings. Stem cuttings with fully developed leaves were transferred to 4% UV-B and 30% UV-A light (18 W, 6500 K) radiation conditions (+). The control corresponds to cuttings not supplemented with UV-B (-). Vertical bars indicate the standard deviations (n=9) and asterisks refer to statistically significant expression changes.

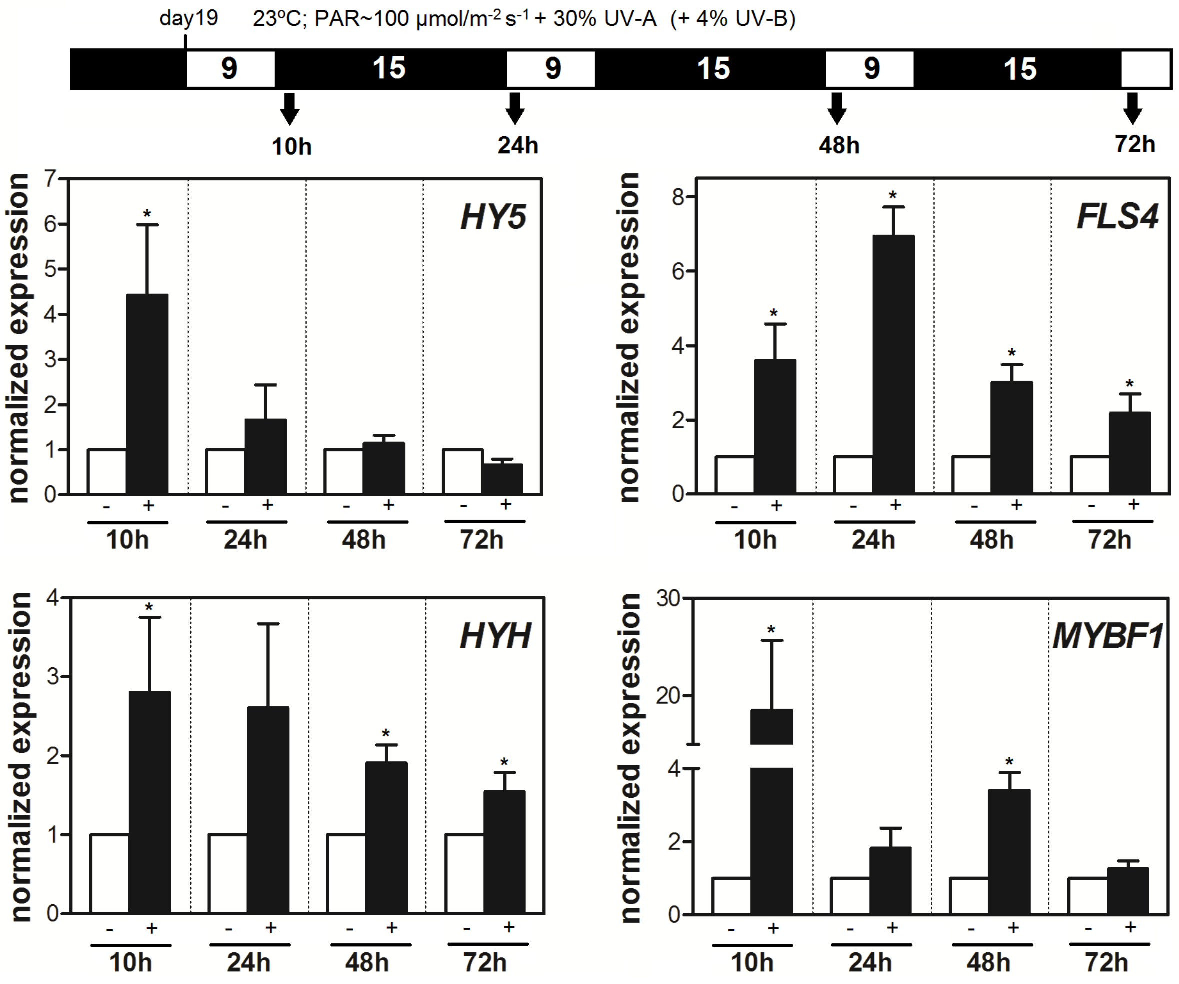

Figure S11

Environmental parameters measured during the UV-B fruit exposure experiments. Temperature, relative humidity and photosynthetically active radiation (PAR) inside the UV-free greenhouse. Measurements taken during the month of January 2013 (A) and on the 15th January (around veraison) from 07.00 h to 23.00 h (B).

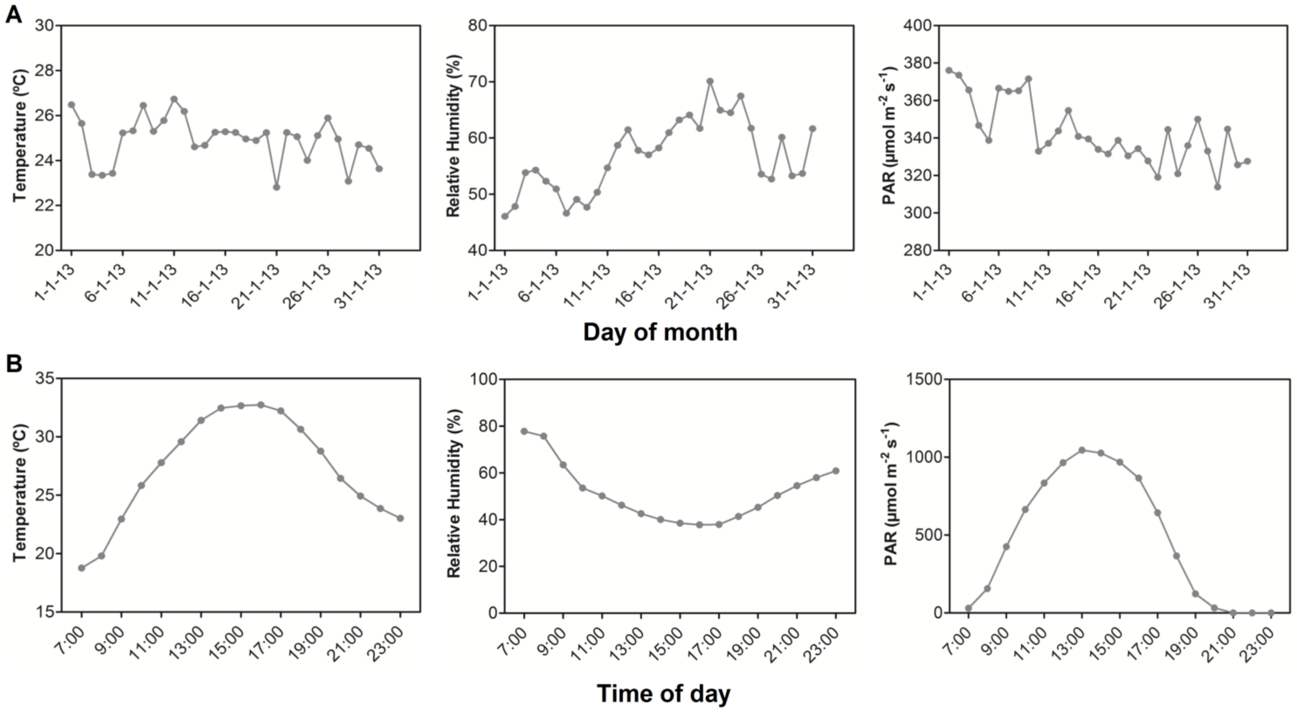

Figure S12

HPLC chromatograms of flavonol analysis for the low UV-B treatment at 9 WAV. Peaks were identified by absorbance pattern inspection, as seen in the box for peak No. 4.

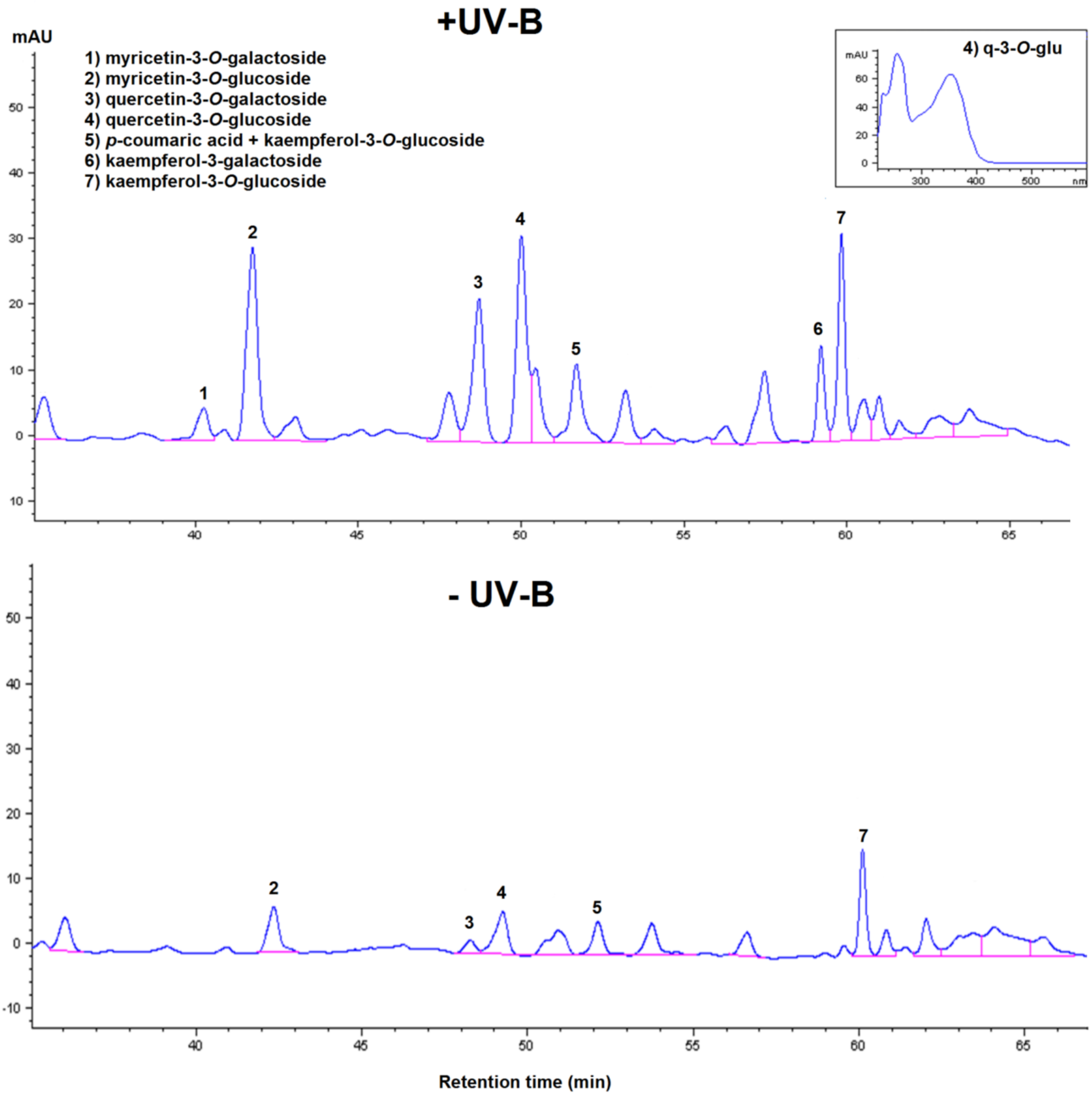

Additional References

Edgar RC. 2004. MUSCLE: multiple sequence alignment with high accuracy and high throughput. Nucleic Acids Research. **32**, 1792–1797.

Hardtke CS, Gohda K, Osterlund MT, Oyama T, Okada K, Deng XW. 2000. HY5 stability and activity in Arabidopsis is regulated by phosphorylation in its COP1 binding domain. The EMBO Journal **19**, 4997-5006.

Osterlund MT, Hardtke CS, Wei N, Deng XW. 2000. Targeted destabilization of HY5 during light-regulated development of Arabidopsis. Nature **405**, 462-466.

Tamura K, Stecher G, Peterson D, Filipski A, Kumar S. 2013. MEGA6: Molecular Evolutionary Genetics Analysis Version 6.0. Molecular Biology and Evolution. **30**, 2725–2729.
